# Polyanhydride Chemistry

**DOI:** 10.1021/acs.biomac.2c01180

**Published:** 2022-11-23

**Authors:** Pulikanti Guruprasad Reddy, Abraham J. Domb

**Affiliations:** School of Pharmacy-Faculty of Medicine, The Hebrew University of Jerusalem, and Centre for Cannabis Research and the Institute of Drug Research, The Alex Grass Centre for Drug Design and Synthesis, Jerusalem 9112002, Israel

## Abstract

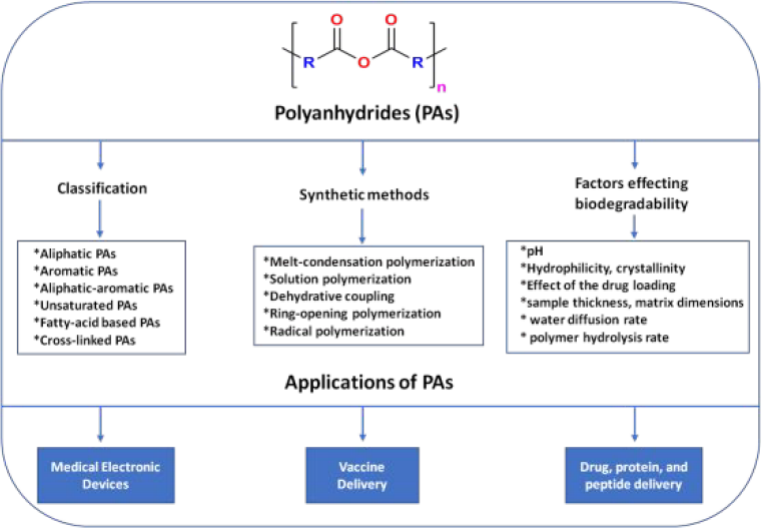

Polyanhydrides (PAs) are a class of synthetic biodegradable polymers employed as controlled drug delivery vehicles. They can be synthesized and scaled up from low-cost starting materials. The structure of PAs can be manipulated synthetically to meet desirable characteristics. PAs are biocompatible, biodegradable, and generate nontoxic metabolites upon degradation, which are easily eliminated from the body. The rate of water penetrating into the polyanhydride (PA) matrix is slower than the anhydride bond cleavage. This phenomenon sets PAs as “surface-eroding drug delivery carriers.” Consequently, a variety of PA-based drug delivery carriers in the form of solid implants, pasty injectable formulations, microspheres, nanoparticles, etc. have been developed for the sustained release of small molecule drugs, and vaccines, peptide drugs, and nucleic acid-based active agents. The rate of drug delivery is often controlled by the polymer erosion rate, which is influenced by the polymer structure and composition, crystallinity, hydrophobicity, pH of the release medium, device size, configuration, etc. Owing to the above-mentioned interesting physicochemical and mechanical properties of PAs, the present review focuses on the advancements made in the domain of synthetic biodegradable biomedical PAs for therapeutic delivery applications. Various classes of PAs, their structures, their unique characteristics, their physicochemical and mechanical properties, and factors influencing surface erosion are discussed in detail. The review also summarizes various methods involved in the synthesis of PAs and their utility in the biomedical domain as drug, vaccine, and peptide delivery carriers in different formulations are reviewed.

## 1 Introduction

Biodegradable polymers have received significant interest in biology and medicine.^1−9^ Based on their origin, these polymers are classified as either natural or synthetic biodegradable polymers.^1^ Examples of natural biodegradable polymers include: proteins, nucleic acids, and polysaccharides (cellulose, starch, and chitosan).^1,3,9^ Most of these polymers are obtained from biological systems or biobased products of vegetable oils, animal fats, and extracts of plant products. Examples of synthetic biodegradable polymers include polyesters,^4^ polyanhydrides,^10−14^ polyphosphazenes,^15^ poly(alkylcyanoacrylates),^16^ poly(amino acids),^17^ and block copolymers with PEG.^18^ These polymers are usually synthesized by condensation, ring-opening and metal-based polymerization reactions with suitable biocompatible organic monomers. Compared to natural polymers, synthetic polymers have attracted more attention in the biomedical research because of their outstanding mechanical properties, biodegradability, biocompatibility, drug/gene loading capacity, and convenience to alter the degradation rates.^1^ Most biodegradable synthetic polymers have been studied for their potential applications in drug delivery (as a controlled drug/gene release reservoir), gene therapy, regenerative medicine, implantable devices, coatings on implants, etc.^1^ A Scifinder search on biodegradable polymers reveals that numerous research articles and patents (more than 10,000) have been recorded since the 1980s.

Controlled release of various therapeutic agents is possible using synthetic biodegradable polymers; where the drug is admixed physically or chemically into the polymer matrix to achieve a suitable oral or injectable drug delivery formulation for sustained release.^1,4,10−18^ The polymer part in the formulations is degraded/eroded at a specific site, and this causes the release of the drug in a controlled manner over time. Generally, synthetic biodegradable polymers consist of a hydrophobic monomer connecting with any of the water labile functional groups such as anhydrides, esters, amides, and imides.^1,4,10−18^ These bonds undergo hydrolytic cleavage or enzymatic cleavage, causing the degradation of the polymer matrix and release of the drug. The degraded products are mostly biocompatible and are easily eliminated from the biological system without exerting any significant adverse effects to the body. Owing to these characteristics, biopolymers have been employed as drug delivery vehicles for a wide variety of low molecular weight drugs, bioactive compounds, macromolecular therapeutic agents, genes, etc.^1,2,4−8,10−18^ Hence, the development of synthetic biodegradable polymers is an active research area in the field of biomedical science.

**Scheme 1:**
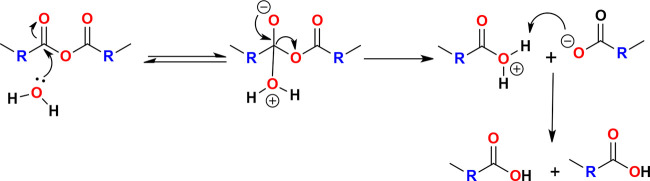
Hydrolytic Cleavage of the Anhydride Bond into Carboxylic Acids

In this context, polyanhydrides (PAs) are attracting much attention for drug delivery applications due to their unique properties, such as controlled biodegradability, zero-order release kinetics for drugs, and low toxicity of the products of degradation.^10−14^ They are highly reactive in aqueous media, resulting in a rapid hydrolytic cleavage to generate acidic monomeric units (Scheme 1). The rate of water penetrating the bulk of the polyanhydride (PA) is much lower than the rate of anhydride bond cleavage occurring at the surface of the polymer. Moreover, the rate hydrolysis of PAs is much higher due to shorter average half-lives than other classes of biodegradable polymers such as polyesters, polycarbonates, and amides.^14^ These characteristics allowed PAs as surface-eroding drug delivery carriers in various forms such as biomedical implantable devices, microparticles, nanoparticles, microspheres, pasty formulations, etc.^10−14,19−23^ By altering the type and ratio of the monomer microstructure, a variety of biocompatible PAs were developed with controlled drug release characteristics and predictable hydrolytic degradation rates.^10−14,19−23^

Development of PAs was first documented by Bucher and Slade in 1909, when they discovered aromatic PAs from the polymerization of isophthalic acid or terephthalic acid in the presence of acetic anhydride at high melting temperatures.^24^ Twenty years later, with an aim of developing PAs for textile applications, Hill and Carothers developed aliphatic PAs based on aliphatic dicarboxylic acid monomer units.^25−27^ Hill synthesized poly(adipic anhydride) from adipic acid by heating with acetic anhydride.^25^ However, these compounds were unsuitable for textile applications, as they underwent hydrolytic cleavage or degradation in room atmospheric moisture. Studies on thermal stability also revealed that these compounds were unstable at higher temperatures due to the formation of cyclic dimers and polymeric rings. Continuing the systematic development of PAs, Conix^28^ and Yodaet al.^29−32^ further developed more than 100 new PAs based on aliphatic and aromatic diacid monomers. These polymers are quite stable toward hydrolytic degradation, and they possess excellent film and fiber-forming properties. Although their discovery led to some progress involving the retention of sustainable thermal and hydrolytic stability associated properties of the PAs upon tuning the composition and monomer choice; however, the commercialization of PAs for use in the textile industry has not been realized yet.

Considering rapid hydrolytic degradation of the PAs as an important characteristic for drug delivery applications, in 1983 Langer et al. exploited the use of these materials for the first time as biodegradable drug delivery carriers.^33^ Following this outstanding invention, exhaustive research has been conducted over the years by several research groups and industries, resulting in numerous research articles and patents.^10−14,19−23,34−38^ The majority of these studies focus on the development of new PA structures, scalable synthetic schemes, physical and chemical characterization, toxicity studies, degradation kinetics, and their applications in the controlled and localized delivery of low/high-molecular-weight therapeutic agents and bioactive compounds. In biomedical applications, a “Gliadel wafer” using poly(sebacic acid-*co*-1,3-bis(p-carboxyphenoxy)propane) (P(CPP-SA)), has been approved by the FDA to deliver the chemotherapeutic agent 1,3-bis(2-chloroethyl)-N-nitrosourea (BCNU) for the treatment of brain cancer.^12,39−41^ However, this is the only PA-based product available commercially in the market. This is probably due to the fact that the handling, storage, and fabrication of PAs is extremely difficult due to their short shelf life’s.^14^ Numerous improvements, though, have been made in PA chemistry to overcome these limitations. One of the significant developments made in this field is the finding of poly(ester-anhydrides).^42−53^ The incorporation of ester into the PA backbone helps to enhance the shelf life of the resultant poly(ester-anhydrides).^44,49,54,55^ In these polymers, the ester-based hydrophobic nonlinear side chain such as hydroxy alkanoic acids shields the anhydride bond. This controls the cleavage of the anhydride linkage from moisture and provides stable polymers. For example, the poly(ester-anhydrides) reported from the monomers of sebacic acid and ricinoleic acid with alternating ester-anhydride bonds is self-stable at RT for 18 months.^44,49^ These polymers are facilitated to use as injectable pasty formulations in the biomedical field due to their viscous and low melting nature.^47,48,54,56^ The viscosity of the pasty poly(ester-anhydrides) depends on their molecular weight distribution. The pasty formulations are easily squeezable from the needle, which can create a deposit under the aqueous atmosphere to release a loaded drug in a controlled manner. This approach is highly useful for localized delivery of drugs with minimal invasion.

**Figure 1:**
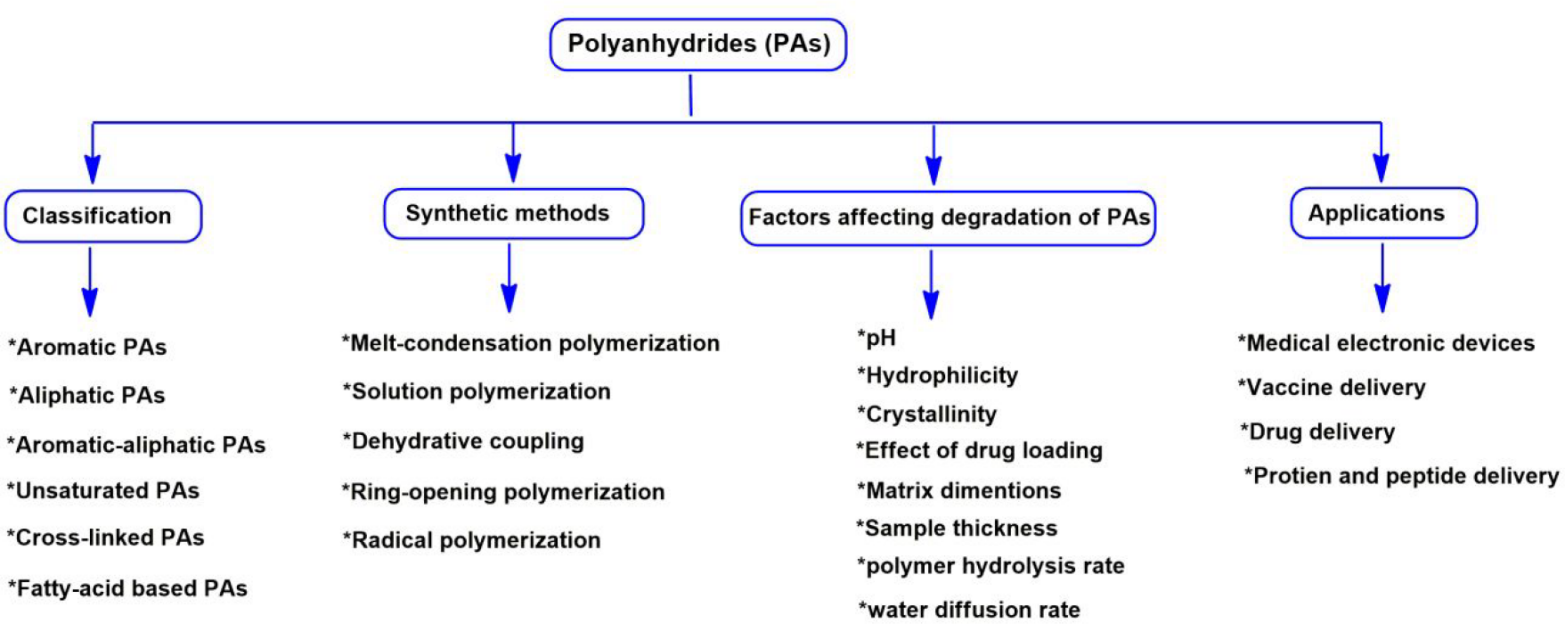
Schematic illustration of the current review focusing on the different classes of PAs and synthetic methods, factors affecting their hydrolytic degradation rates, and their applications in the biomedical field.

Considering the above, the present review highlights the chemistry of different classes of PAs and synthetic methods, factors affecting degradation of PAs, and their applicability as drug delivery carriers for vaccine, drug, and proteins as well as their potential utility in the bioelectronics have been discussed predominantly (Figure 1). We believe that the fundamental and advanced information described in the present review will help readers to handpick the advanced PA based drug delivery carriers in the biomedical field as well as to design of novel biocompatible PAs with high biomedical utility.

## 2 Classification

PAs are classified into aromatic, aliphatic, and unsaturated PAs.^10−14,19−23,34−38^ However, owing to the use of PAs for biomedical applications, other subclasses such as aromatic–aliphatic, cross-linked, and fatty-acid based PAs have also been developed. The classification of PAs is mainly based on the type of monomer unit connected through an anhydride bond. In this section, we outline the chemistry, thermo-mechanical properties, drug loading, and erosion kinetics of various PAs. They are discussed with selected examples.

### 2.1 Aromatic Polyanhydrides

**Scheme 2:**
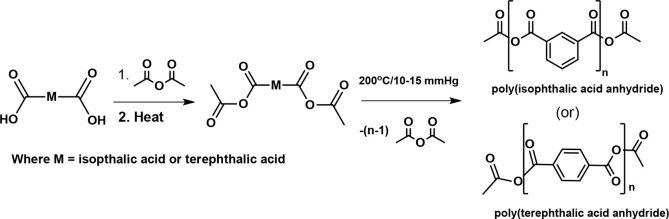
Synthesis of Poly(isophthalic anhydride) and Poly(terephthalic anhydride) through Melt-Condensation Polymerization

Aromatic PAs were prepared by melt condensation in the presence of acetic anhydride under reflux conditions. For example, the synthesis of poly(isophthalic anhydride) and poly(terephthalic anhydride) has been provided in Scheme 2.^24^ A gamut of aliphatic, aromatic, unsaturated PAs, and combinations thereof have been developed by several research groups employing the method depicted below.^10−14,19−23,34−38^ This is currently the most widely employed method for the synthesis of PAs.^13^

Aromatic PAs possess high mechanical strength and hydrolytic stability that slows down their degradation thereby, making them useful candidates for sustained drug release applications. Representative aromatic PAs are shown in Table 1. Most aromatic PAs are insoluble in common organic solvents and possess high melting points, usually above 200 °C. These features create issues during the fabrication of PAs into films or microspheres using solvent or melt techniques.^57^ The aromatic monomers are copolymerized with aliphatic monomer units to attain necessary physicochemical characteristics. A series of aromatic–aliphatic PAs made from isophthalic acid (IA), terephthalic acid (TA), 1,3-bis(*para*-carboxyphenoxy)-propane (*p*-CPP), 1,6-bis(*para*-carboxyphenoxy)hexane (*p*-CPH), and aliphatic sebacic acid (SA) have been developed with improved thermo-mechanical characteristics.^10−14,19−23^

**Table 1:**
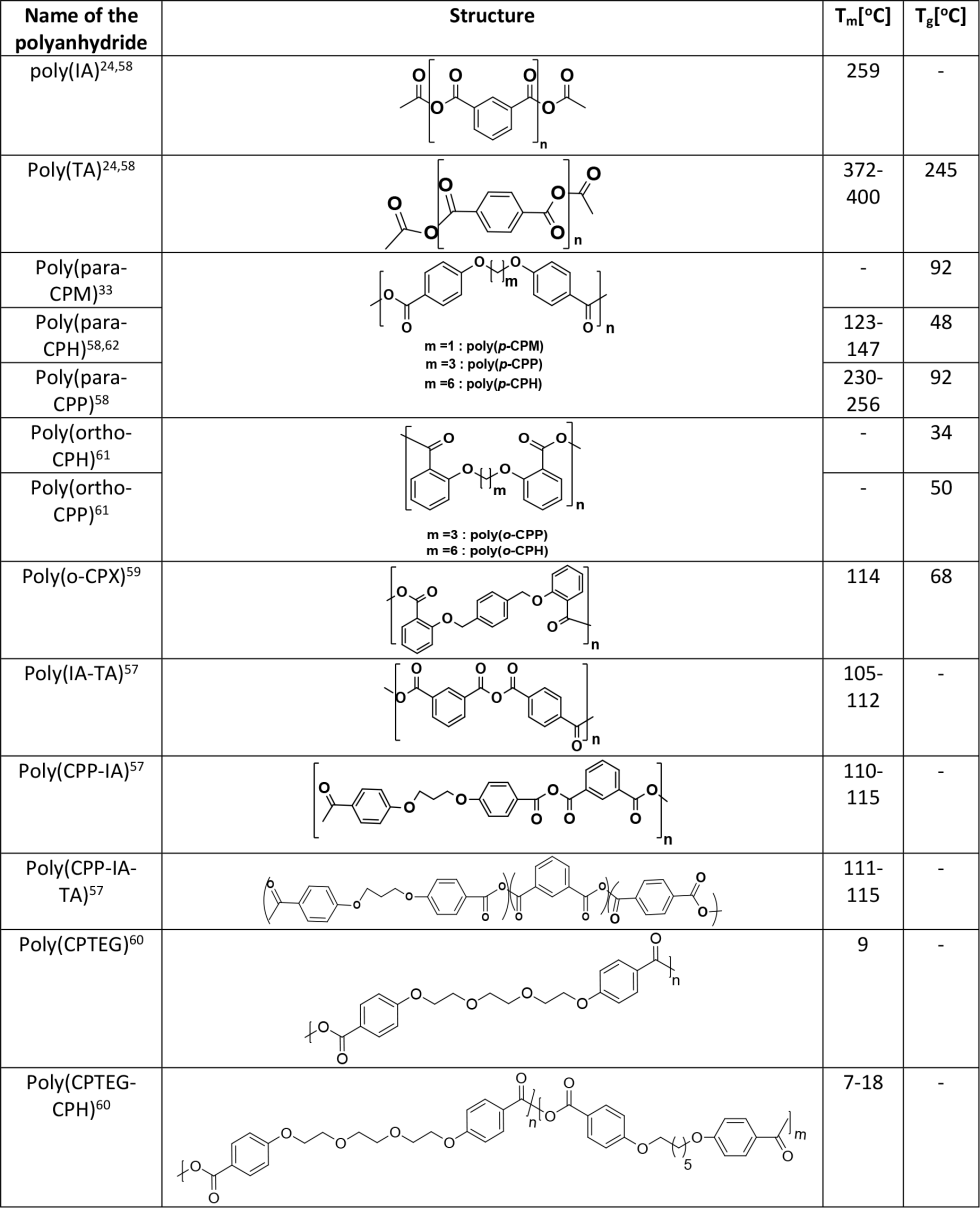
Aromatic Polyanhydrides

The aromatic PAs, poly(*p*-CPH) (n = 6) and poly(*p*-CPP) (n = 3) and their copolymers have been synthesized by melt condensation polymerization of the corresponding dicarboxylic acid monomers with acetic anhydride.^58^ The degradation rate of these polymers increases with the increase of methylene carbon chain length in the polymer backbone. The degradation of these polymers is pH dependent.

In 2014, Uhrich et al. reported another poly aromatic biomaterial, poly[α,α′-bis(*ortho*-carboxyphenoxy)-paraxylene] poly(*o*-CPX) (Table 1). They investigated its potential as a drug delivery matrix with respect to the study of the hydrolytic degradation, radiation stability, and biocompatibility of the polymer.^59^ Poly(*o*-CPX) possesses excellent solubility in common organic solvents, and its *T*_g_ (68 °C) falls above the physiological temperature, also within a practical range of thermal processability. Poly(*o*-CPX) disks show surface erosion over a period of 6 months. HPLC and UV analyses reveal that the polymer is degraded to release diacid precursors into the buffer solutions. Cytotoxic experiments conclude that the degraded products of the polymer are biocompatible.

Narasimhan and co-workers developed homo/co-PAs i.e., poly(CPTEG) and poly(CPTEG-CPH)(Table 1) based on the aromatic monomer units of CPH, and 1,8-bis(p-carboxyphenoxy)-3,6-dioxaoctane (CPTEG).^60^ The polymers were synthesized through the melt-condensation polymerization process. The polymers possess low glass transition temperature (9–18 °C) and do not show any melting temperature. Poly(CPTEG-CPH) is amphiphilic, whereas the CPTEG segment is presented within the CPH hydrophobic backbone. The erosion behavior of this polymer is fine-tuned from the bulk-erosion to surface erosion upon increasing the CPH content due to the increase of polymer crystallinity. Crystalline polymers erode more slowly than amorphous polymers. For example, poly(CPTEG) showed a faster erosion mechanism that deviates from the surface erosion mechanism due to the lack of crystallinity. The applicability of one of the copolymers, i.e., poly(CPTEG-CPH) (20:80) is highly compatible with the delivery of a variety of vaccines and proteins. The advances made in the biomedical field using this polymer are summarized in the Applications section.

Changing the substitution pattern of the phenyl ring and increasing alkyl chain length influence the change of aromatic PA properties. The thermo-mechanical properties of aromatic PAs are influenced by the position of the polarizable reactive functional group residing in the monomer structure.^57^ For example, poly(TA) showed glass transition (*T*_g_) and melting temperatures (*T*_m_) at 245 and 372 °C, while its isomeric poly(IA) showed lower *T*_m_ at 259 °C. This is due to disruption in the periodicity of IA caused by the polymerizing reactive functional group (carboxylic acid) situated in the meta-position of the phthalic acid. Similarly, the *T*_m_ of poly(TA-IA)) obtained from IA and TA were seen between 105–112 °C, which is lower than the *T*_m_ of poly(TA) or poly(IA). Inclusion of IA units leads to higher-order disruption of the structural periodicity in poly(TA-IA). The crystalline melt temperatures of the aromatic PAs are also influenced by the alkyl chain length in the main chain of the polymer. For example, poly(CPP) and poly(CPH) showed *T*_m_ at 256 and 123 °C. Increases in alkyl chain length from propyl to hexyl in the case of poly(CPH) lead to potentially large differences in the polymer thermal and crystalline properties. Similarly, to improve the solubility and *T*_g_ of the poly(*p*-CPP) and poly(*p*-CPH), in 1999, Uhrich et al. reported analogue ortho derivatives such as poly[1,3-bis(*o*-carboxyphenoxy)propane anhydride] poly(*o*-CPP) and poly[1,6-bis(*o*-carboxyphenoxy)hexane anhydride] poly(*o*-CPH) (Table 1) by shifting the substitution of the phenyl ring pattern from para to ortho.^61^ By changing the aromatic substitution pattern from para to ortho and lengthening the alkyl chain length between the aryl groups from propane to hexane, they achieved enhanced solubility for poly(*o*-CPP) and poly(*o*-CPH) with lowered *T*_g_ values.^61,62^

### 2.2 Aliphatic Polyanhydrides

Aliphatic PAs are synthesized from the saturated diacid monomer units through a melt-condensation process. The synthesis involves the formation of an adipic anhydride monomer initially from the heating of adipic acid with acetic anhydride, followed by ring-opening polymerization under reflux/vacuum conditions, yielding poly(adipic anhydride).^25^ Further, Carothers and Hill co-workers reported further another synthetic aliphatic PA “poly(sebacic anhydride) (poly(SA))” using sebacic acid as a starting monomer under similar reaction conditions.^26^ Aliphatic PAs possess low melting (*T*_m_) and glass transition temperatures (*T*_g_) compared to other classes of aromatic or aromatic–aliphatic PAs.^10−14,19−23^ This is due to the high mobility of the aliphatic chain. This cannot be seen in aromatic PAs, as they show rigidity due to potential π–π stacking interactions between the aromatic molecules. The aliphatic PAs are crystalline, melt at a temperature below 100 °C, and are soluble in chlorinated hydrocarbons. For example, poly(SA) shows a *T*_m_ of 88 °C and a *T*_g_ below ambient temperature.^63^ These are biodegradable and eliminated from the body within a span of weeks.^64^ These properties allow the use of aliphatic PAs for short-term biomedical applications. For clarity, a few representative examples of common aliphatic PAs employed in the biomedical sector and their thermal parameters are shown in Table 2.

**Table 2:**
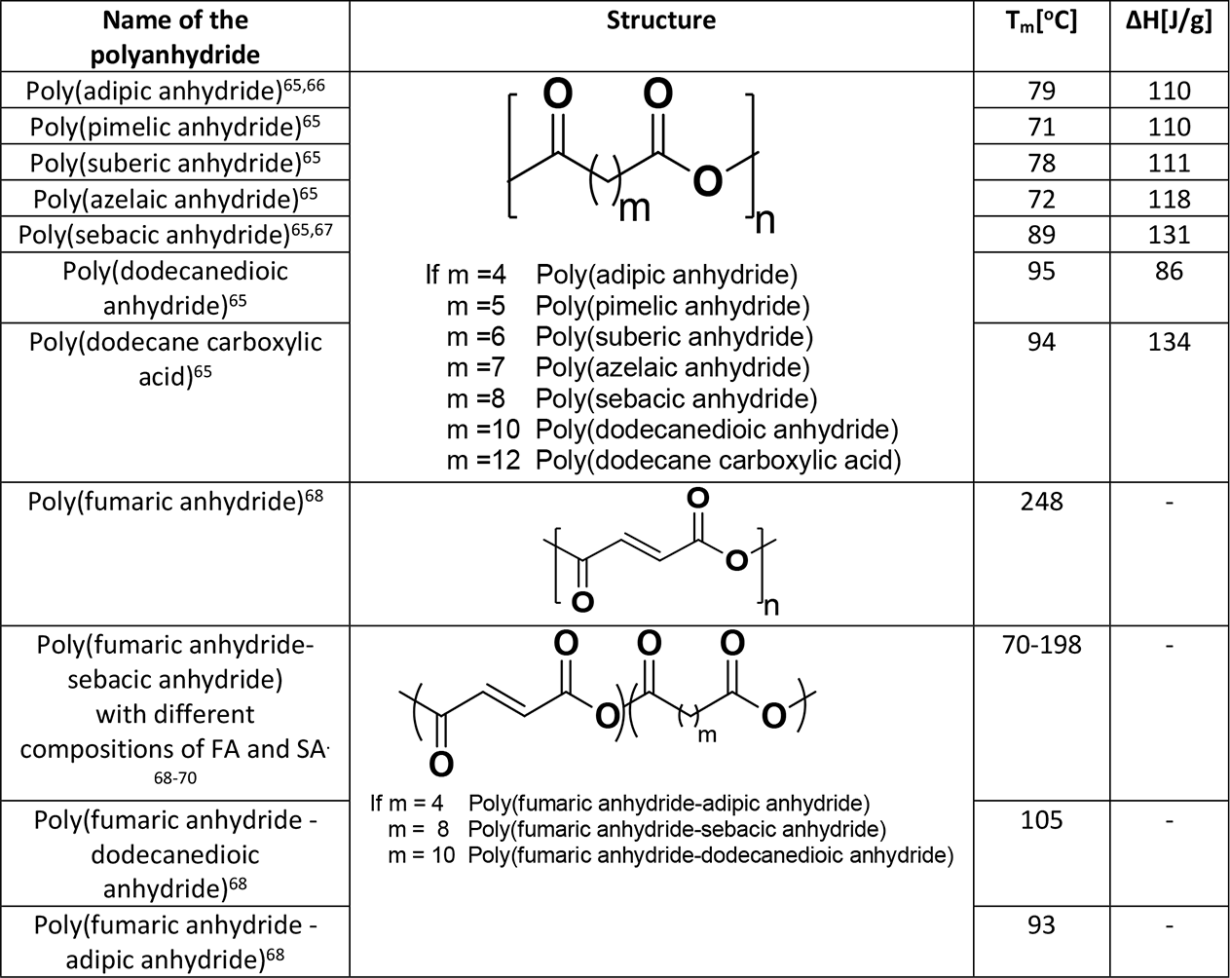
Aliphatic and Unsaturated Polyanhydrides

The hydrolytic degradation of aliphatic PAs is altered by a simple change in the structure of the polymer backbone or increasing hydrophobic content in the polymeric structure. A series of high molecular weight aliphatic PAs based on aliphatic diacid monomers such as sebacic acid, adipic acid, and dodecanedioic acid have been reported.^64,65^ Using a variety of metal catalysts including cadmium acetate, ZnEt,-H_2_O(1:1), barium oxide, calcium oxide, and calcium carbonate, high molecular weight PAs in the range of about 245,000 were synthesized. These polymers are rigid and crystalline and show high melting temperatures which are proportional to the monomeric chain lengths. Rectangular drug delivery matrices (3 × 7 × 11 mm^3^) fabricated from azelaic, sebacic, and dodecanedioic PAs, showed surface erosion of about 20% in 48 h. In contrast, drug delivery matrices composed of PAs based on the short-chain monomers (adipic, pimelic, and suberic) showed nearly about 70% of mass loss in the same timeline.

The properties of aliphatic PAs are also strongly influenced by their molecular weight and molecular weight distributions. Therefore, it is essential to maintain the controlled and reproduced molecular weights of the polymer for medical usage. Therefore, our group studied the proof of concept to get the controlled molecular weight of the aliphatic PAs (poly(suberic acid), poly(azelaic acid), poly(sebacic acid), and poly(dodecanedioic acid)) with varying acetic anhydride concentrations.^55^ An increase in the polymer molecular weight is dependent on the concentration of the acetic anhydride used. At higher acetic anhydride concentrations (1 equiv), the polymer is obtained with high molecular weights and a good polydispersity index. At lower acetic anhydride concentrations (<1 equiv), the leftover carboxylic acid monomers in the reaction mixture act as chain terminators; thus, the formation of the lower molecular weight polymers.

However, taking into consideration their high propensity for hydrolytic degradation, these polymers have been explored for biodegradable drug delivery systems. For example, Albertsson et al., developed microsphere-gel ocular drug delivery formulations based on poly(adipic anhydride) for controlled release of timolol maleate.^66^ The drug formulations were prepared through a nonaqueous solvent removal technique. The polymer is degraded through the surface erosion. The incorporated drug was released from the polymeric microspheres in a controlled manner. Similarly, amphiphilic block copolymer microspheres prepared from poly(SA) and Pluronic-F68/F127 have been used for the sustained delivery of nifedipine.^67^ Recently, the poly(SA) nanoparticles for the controlled nasal delivery of thyrotropin-releasing hormone (TRH)^56^ has been reported. TRH-loaded nanoparticles are prepared by the solvent-anti solvent process under anhydrous conditions. Most of the TRH was released from the nanoparticles within an hour in the water as a release medium. The nanoparticles are less toxic at lower concentrations as revealed by concentration-dependent cell toxicity studies. In addition, the poly(SA) also used as a carrier to study the *in vitro* release pattern of the anticancer drug Temozolomide (TMZ) in the acetate buffer solutions (pH = 3.5) at 37 °C.^55^

Toward the development of novel aliphatic PAs for biomedical applications, the recent highlights made in this section are further summarized as follows:

#### 2.2.1 Covalent Insertion of Cyclic Oligosaccharides into the Cyclic-Polyanhydride Backbone

The incorporation of cyclic oligosaccharides into the PA backbone is an interesting strategy to enhance the oral bioavailability of drugs. The cyclic oligosaccharides act as a host, which can load the guest drug molecules in its cavity. For example, the nanoparticles of cyclodextrin (CD)-linked conjugate polymer (CD-PVM/MA) (PVM/MA = poly(methyl vinyl ether-*co*-maleic anhydride)) enhances the oral bioavailability of several drugs such as camptothecin,^71^ paclitaxe,^72^ and atovaquone.^73^ The CD-PVM/MA copolymer was obtained through an esterification reaction between hydroxyl propyl-β-cyclodextrin and poly(PVM-MA).^74^ Recently, Lucio et al. used the nanoparticles of CD-PVM/MA to enhance the oral administration of the anti hyperglycemic agent “glibenclamide”.^74,75^ The drug-loaded nanoparticles are tested for their hypolipidemic effect in a C. elegans model. Similarly, Demirel et al. reported the use of rosuvastatin calcium incorporated CD-PVM/MA nanoparticles to enhance the poor oral bioavailability of rosuvastatin calcium.^76^ Pharmacokinetic studies conclude that the approximately 8-fold relative oral bioavailability enhancement is achieved in the case of rosuvastatin calcium-loaded CD-PVM/MA nanoparticles compared to the pure rosuvastatin calcium.

#### 2.2.2 Covalent Insertion of Bioactive Drugs into the Aliphatic Polyanhydride Backbone

**Scheme 3:**
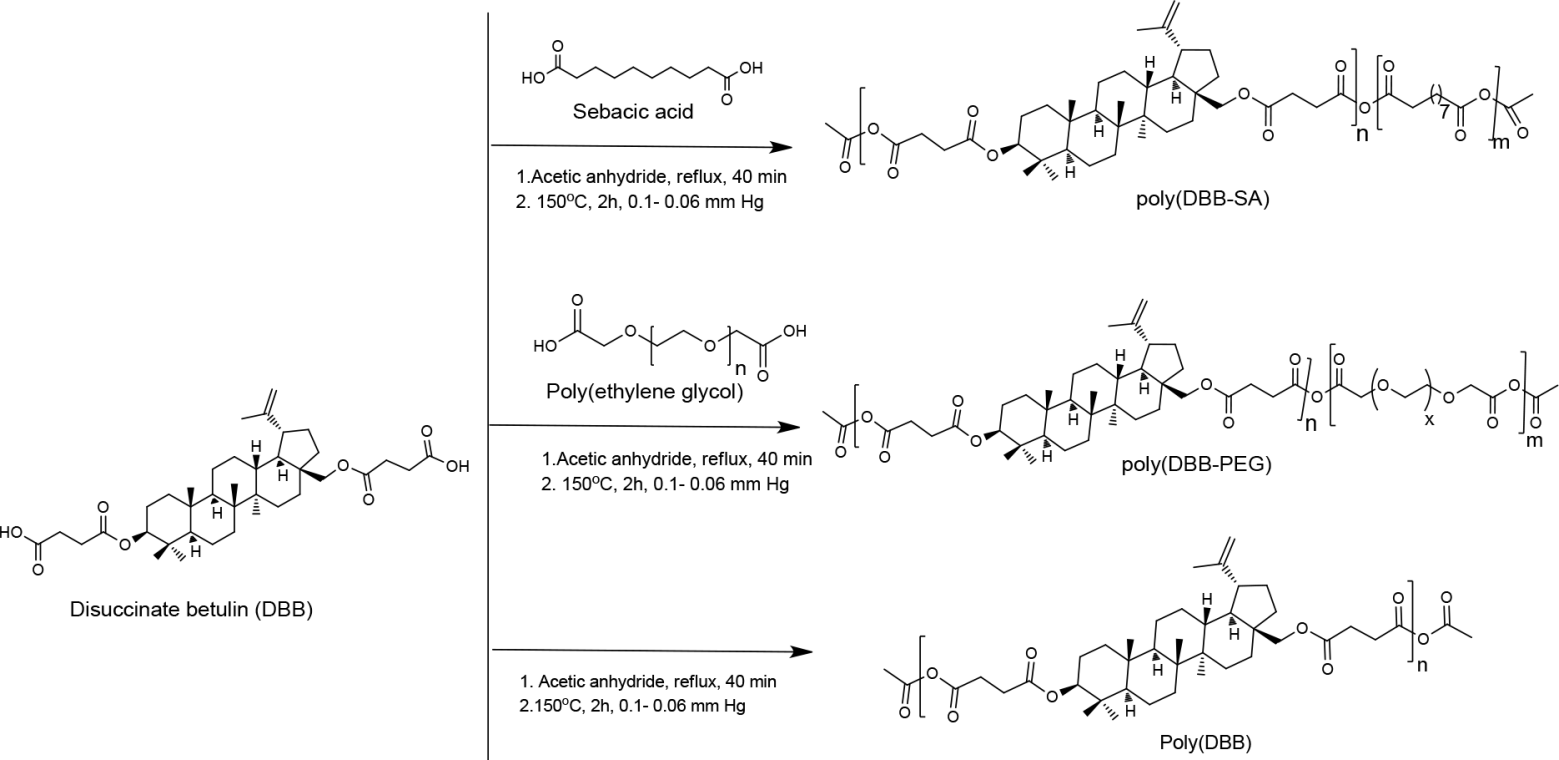
Synthetic Route of Poly(DBB), Poly(DBB-PEG), and Poly(DBB-SA) from the Melt-Condensation Polymerization of Disuccinate Betulin (DBB) and Sebacic Acid (or) Polyethylene Glycol (PEG) Used with permission of Royal Society of Chemistry, from Novel Polymeric Derivatives of Betulin with Anticancer activity, Niewolik, D.; Krukiewicz, K.; Bednarczyk-Cwynar, B.; Ruszkowskic, P.; Jaszcz, K., Vol. 9, 2019; permission conveyed through Copyright Clearance Center, Inc.

The development of drug-bearing PA or insertion of the drug as a pendant group into the PA chain is another approach for the design of biomaterials. In these polymers, the direct release of the bioactive drugs occurs at the targeted specific site after the degradation of the anhydride bond. The bioactive drugs containing carboxylic acid groups are excellent raw materials for synthesizing such PA pro-drugs. In some cases, the dial-containing drugs are also used as starting materials after converting them into dicarboxylic acids. For example, Jaszcz et al. developed betulin-bearing PA prodrug poly(DBB) through the melt condensation polymerization of the disuccinate betulin, which is obtained by the esterification of betulin with succinic anhydride (Scheme 3).^77^ This polymer in the form of micro and nanoparticles is highly potential for anticancer effects, as they undergo hydrolytic cleavage in the physiological conditions for the sustained release of “disuccinate betulin” completely in 14 days.^77,78^ Further, the release rate of succinate betulin from the poly(DBB) is improved by the incorporation of other comonomers of dicarboxylic derivatives such as sebacic acid^79^ and poly(ethylene glycol)^80^ in the copolymer chain through melt-condensation copolymerization (Scheme 3). Sebacic acid improves the crystallinity of poly(DBB), as a result, faster and controlled release of the succinate betulin occurs from the poly(DBB-SA) copolymers within a span of 5 days. In one of their studies, the poly(DBB-SA) copolymer microspheres are used for the controlled delivery of rifampicin (RIF), which is an ansamycin drug used in the treatment of tuberculosis.^81^ On the other hand, the covalent incorporation of PEG into the poly(DBB) lowers the *T*_g_ of the poly(DBB), thus improving the elasticity. PEG also improves the solubility and hydrolytic degradation properties of the poly(DBB) in addition to controlling the morphology and internal structures of poly(DBB) microspheres. Compared to the poly(DBB) and poly(DBB-SA) copolymers, the PEG-containing poly(DBB) samples are undergoing strong hydrolytic cleavage within a day and release the active drug succinate betulin. All these polymers are reported to show inhibition of the cancer cell growth with minimal cytotoxic effects.

#### 2.2.3 Pegylated Aliphatic Poly(anhydride) Nanoparticles

The poor oral bioavailability of chemotherapy drugs such as taxanes (ex: docetaxel and paclitaxel) can be improved by using pegylated poly(anhydride) nanoparticles as drug delivery carriers. Pegylation of poly(anhydride) nanoparticles slightly decreases the mean size and negative zeta potential of the nanoparticles. Pegylation allows high amounts of docetaxel or paclitaxel to load into the nanoparticles core. Recent articles by Ruiz-Gatón et al. prepared the docetaxel loaded pegylated poly(anhydride) nanoparticles by the incubation between the copolymer of methyl vinyl ether and maleic anhydride, poly(ethylene glycol) (PEG-2000 or PEG-6000), and docetaxel.^82,83^ The oral administration of this docetaxel loaded pegylated poly(anhydride) nanoparticles into mice leads to sustained and prolonged docetaxel availability in the plasma levels over a period of about 3 days.

### 2.3 Unsaturated Polyanhydrides

Unsaturated PAs are synthesized from the aliphatic or aromatic monomers, consisting of unsaturated double/triple bonds. A series of unsaturated PAs based on the monomers of fumaric acid, 2-butynedioic acid, acetylene dicarboxylic acid (ACDA), or 4,4′-stillbenedicarboxylic acid (STDA) and their copolymers with SA prepared by melt condensation or solution polymerization methods have been reported.^68^ These PAs, containing double/triple bonds in the backbone are available for secondary polymerization to form a cross-linked structure with improved mechanical and physical properties. Unsaturated PAs are crystalline and are insoluble in common organic solvents. These polymers also showed high melting temperatures (Table 2). The solubility and thermal properties of these materials were fine-tuned by copolymerization with aliphatic monomer units. For example, the copolymers of fumaric acid with SA., i.e. poly(FA-SA), showed better solubility in chlorinated solvents than poly(FA), which is insoluble in common organic solvents. The copolymers also showed a decrease in melting temperatures, which was associated with an increase of the aliphatic content in the copolymer backbone.

Some unsaturated co-PAs have been developed for special biomedical applications. For example, poly(fumaric acid-*co*-sebacic acid) p(FA-SA) derived from fumaric acid and sebacic acid was used in bioadhesive oral delivery systems that interact with the mucosal tissue.^69^ Degradation of poly(FA-SA) microspheres made up of various FA:SA compositions (20:80, 50:50, and 70:30) was studied *in vitro* at pH = 4.2, 7.4, and 8.8.^84^ The degradation rate of the microspheres under basic conditions (pH = 8.8) was significantly higher than those seen in neutral (7.2) and acidic (4.2) media. Another factor influencing the degradation rate of PA-based microspheres is additive loading. For example, p(FA-SA) (20:80) microspheres loaded with 2% of bovine serum albumin exhibited an accelerated polymer degradation rate.^70^ Various unsaturated PAs and their copolymers reported for biomedical applications are shown in Table 2.

### 2.4 Cross-Linked Polyanhydrides

Cross-linked PAs are usually synthesized by the melt-condensation or photopolymerization of monomers, consisting of anhydride bonds with the unsaturated end-caps.^10−14,19−23^ Cross-linking is an effective method to tailor the physical, mechanical, and degradation properties of PAs. PAs prepared by this method offer high mechanical properties, good thermal stability, and resistance to solvent evaporation.^85,86^ Various cross-linked PAs developed for orthopedic fixation devices (pins, screws for bone augmentation and regeneration, bone cement, etc.), and drug delivery applications are summarized in Table 3.

**Table 3:** Cross-Linked Polyanhydrides Reported for Biomedical Applications^a^

cross-linked polyanhydride	application	ref
Cross-linked poly(anhydride-imides). Examples:poly(TMAala-SA), poly(TMAgly-SA)	Release of the model drug p-nitroaniline	(97)
Cross-linked poly(ester-anhydrides) made-up withpoly(ε-caprolactone) and succinic anhydride	Sustain release of propranolol:HCl	(98)
cross-linked poly(ether-anhydrides) Example: poly(TMPTA)	Sustain release of paclitaxel	(99)
cross-linked poly(thioether anhydrides) Example: poly(PETMP–PNA)	cellular delivery of cytotoxin Hoechst 33342; release of hydrophilic dye (acid orange and AO8); release of marcaptopurine and lidocaine	(94, 95, 89)
cross-linked methacrylatedpolyanhydrides Example: poly(MSA); Poly(MCPP); Poly(MCPH), and poly(MCPH-MSA) incorporated with Si and Sr doped hydroxyapatite-collagen nanoparticles	orthopedic fixation devices; bone regeneration	(96, 100−102)

a TMAala = N-trimellitylimido-*b*-alanine; TMAgly= N-trimellitylimido-glycine; TMPTA = trimethylolpropane triacrylate; PETMP= Pentaerythritol tetrakis(3-mercaptopropionate); PTE = Pentenoic anhydride; MSA = methacrylated sebacic anhydride; MCPH = methacrylated 1,6-bis(carboxyphenoxy) hexane; MCPP = methacrylated1,3-bis(p-carboxyphenoxy) propane.

Few recent advances made in this topic are summarized as follows: Shipp et al. reported the cross-linked poly(thioether anhydride) based on the radical medicated photopolymerization between the starting monomers of tetrathiol, dithiol, and 4-pentenoic acid.^87−91^ They have obtained both amorphous and semicrystalline poly(thioether anhydrides) based on varying enes: thiols stoichiometry. The synthetic details for poly(thioether anhydrides) are discussed in the radical mediated photopolymerization methods. These polymers are largely undergoing surface erosion. The degraded products from these polymers are less toxic. Geraili et al. studied the factors influencing the degradation rate of poly(thioether anhydrides).^92^ Various factors influence the erosion of the cross-linked polymer including the stoichiometry of the starting monomers, temperature, pH of the media, the geometry of the polymers, the media shaking rate, etc. Lowering the cross-linking density of the poly(thioether anhydride) allows faster erosion. This can be achieved by increasing the dithiol monomeric content in the polymer. The erosion behavior of the poly(thioether anhydride) is accelerated by increasing the temperature, surface area of the polymer geometry, pH of the release medium, and media shaking rate. In addition, the shape of the polymer geometry also influences the erosion rate. For example, the poly(thioether anhydrides) in the form of a small cylindrical shape is shown to be a higher erosion profile due to reduced mass when compared to the polymer with a large cylindrical shape. Therefore, this class of polymers maintains surface erosion behavior even in small dimensions; hence, they can be used as small-sized drug delivery systems.

The hydrolytic degradation properties of the cross-linked poly(thioether anhydride) disks immersed in the phosphate buffer solution at different time points are studied using Raman spectroscopy.^93^ The hydrolytic degradation properties were monitored spatially and temporally via Raman kinetics studies at various depths of penetration into the sample. The studies conclude that the cross-linked poly(thioether anhydride) is indeed undergoing surface erosion but the degradation also starts to occur in the core of the sample at a shorter time. The percentages of anhydride bonds that remain in the sample are decreased with the degradation time in all the depths, however, the degradation is much faster in the edges of the sample as compared to the center.

The applicability of the amorphous poly(thioether anhydrides) is explored as drug-release carriers for the release of both hydrophilic and hydrophobic drugs such as 6-marcaptopurine and lidocaine *in vitro* conditions.^94^ The release of the anticancer drug “6-marcaptopurine” from the polymer matrix is influenced by the diffusional behavior of the drug as well as the degradation process of the polymer. The release of aesthetic lidocaine is affected by polymer degradation rather than by diffusion. Another study by the same group reported the cellular delivery of the Hoechst 33342 anticancer drug from the poly(thioether anhydrides).^95^ The released drug from the polymer matrix shows the change in the cell morphology and leads to cancer cell death.

The mechanical strength of the cross-linked PAs can improve doping with nanoparticle composites. For example, Sudip et al. reported the hydroxyapatite-collagen nanoparticles incorporated cross-linked PA pasty formulations for the bone regeneration capacity *in vitro.*(96) The PA-nanoparticle blends were synthesized based on the radical medicated photopolymerization between the monomers of methacrylated SA and CPH in the presence Si and Sr doped hydroxyapatite-collagen nanoparticles. Blending of the nanoparticles to the extent of 10 wt % into PA matrix improves the compressive strength of the hardened paste from 30 to 49 MPa. The metal doped nanocomposites in the cross-linked polymer also improve the osteogenic capacity of self-flowable paste, which is suitable for the resurrection of complex shaped musculoskeletal defects.

### 2.5 Aromatic–Aliphatic Polyanhydrides

Aromatic–aliphatic PAs have been developed based on the copolymerization of aromatic and aliphatic monomers through melt condensation.^10−14,19−23^ These polymers possess physiochemical characteristics such as better mechanical strength, controlled degradation/erosion rate, melting temperatures, and solubility, which are needed for biomedical purposes. These polymers are semicrystalline in nature, and their degree of crystallinity is lower than aromatic PAs. The mechanical and thermal properties of these polymers are improved compared to aliphatic PAs.

**Figure 2:**
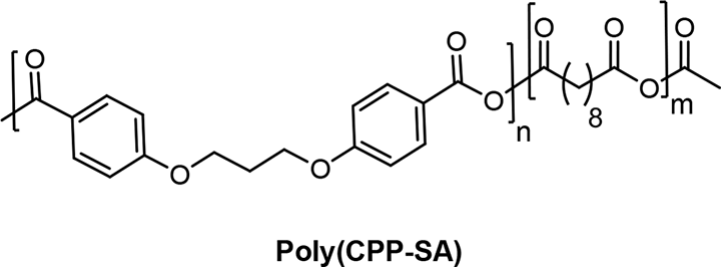
Chemical structure of the poly(CPP-SA).

One of the classic examples reported in the class of aliphatic-aromatic PA is poly[CPP-SA] (Figure 2). The polymer is prepared by the melt copolymerization between the starting monomers of SA and CPP.^58^ The high CPP content extends the erosion rate and tensile strength of the poly(CPP-SA).^65^ Recently, poly(CPP-SA) microspheres are being used as delivery carriers for the release of both hydrophilic and hydrophobic drugs.^103^ The poly(CPP-SA) microspheres with core/shell-like structures are synthesized based on the water-in-oil-in-water double emulsion and solvent evaporation methods. The microspheres can encapsulate both hydrophilic and hydrophobic drugs in their core and shell during the fabrication process. Brilliant Blue G and curcumin are encapsulated as model hydrophilic and hydrophobic drugs in the poly(CPP-SA) microspheres with an encapsulation efficiency of about 40–45% and 90%, respectively. The microsphere shell acts as a barrier that can control the release rate of the hydrophilic drug from the microsphere core. Initially, the controlled release of the hydrophobic drug occurs from the microsphere shell, followed by the release of the hydrophilic drug from the microsphere core. The release rate of the hydrophobic drug is extended by the increases in aromatic CPP content in the copolymer.

**Scheme 4:**
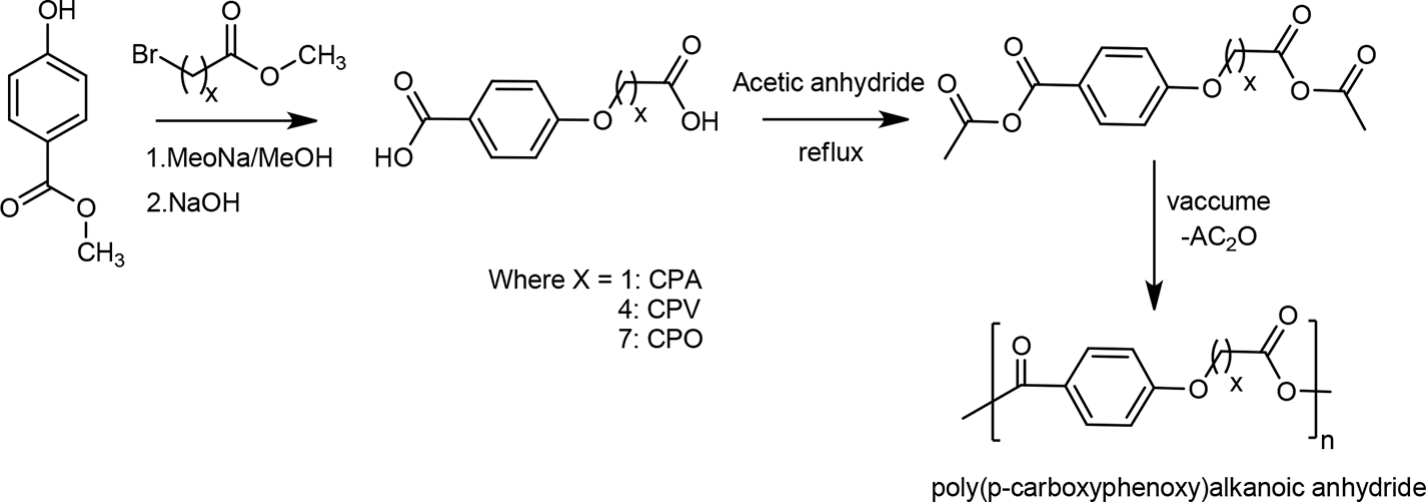
Synthetic Route for Poly(p-carboxyphenoxy)alkanoic Anhydrides Reproduced with permission from ref (104), Copyright 1989 American Chemical Society.

To develop novel PA copolymers with more linear release profiles, homo and co-PAs of carboxyphenoxy alkanoic acids were synthesized (Scheme 4).^104^ The monomeric units were generated by combining both aromatic-acid and aliphatic-acid units in one chemical entity through a nonlabile bond. Increase in the alkanoic chain length slower the degradation rate of the polymer. The degradation rate of various copolymers based on the increase of alkanoic chain length is as follows: poly(CPA) > poly(CPV) > poly(CPO). These polymers showed very good stability in the solid state over 6 months under anhydrous conditions at 25 °C. Further, a series of aromatic–aliphatic PAs based on the melt copolymerization of IA, TA, and CPP monomers with fumaric acid or sebacic acid is reported.^57^ The aim of the study is to improve the physicochemical properties of poly(IA), poly(TA), and poly(CPP) by introducing aliphatic/unsaturated diacids into the polymer chain. The copolymers of poly(CPP-FA) containing 10–60 mol % of CPP are amorphous. These polymers are melting at below 120 °C compared to copolymers with more CPP content (more than 60 mol %). An increase in CPP content increases the crystalline and melting properties of the copolymer. Similarly, the copolymers of fumaric acid containing either 15–40% of TA or 10–90% of IA showed low melting temperatures than compared to poly(fumaric acid). Adding a third monomer into the copolymer structure further decreases melting, and crystalline properties and greatly enhances the solubility properties of the polymer. For example, the terpolymers, i.e., poly(CPP-IA-SA), poly(TA-IA-SA), and poly(TA-CPP-SA) containing 10–25% of SA are pliable, and melt at low temperatures compared with the copolymers made-up with CPP or IPA or TA and 30 mol % of SA.

### 2.6 Fatty Acid-Based Polyanhydrides

Incorporating fatty acid monomers into the PA backbone improves characteristics such as flexibility, hydrophobicity, and pliability.^10−14,19−23^ The degradation of these polymers produces naturally occurring fatty acids that are nontoxic to the biological environment. The fatty acid monomers are incorporated into the polymer matrix in the following two ways: (1) monofunctional fatty acids as chain terminators during PA synthesis, (2) conversion of these monofunctional fatty acids into dimers for that are then utilized in the PA synthesis. Dicarboxylic fatty acids are usually obtained by the dimerization of fatty acids through the unsaturation of double bonds or by the incorporation of the additional carboxylic acid side chains on the hydroxyl group of the fatty acids through an esterification process. The structures of a few mono and dimer fatty acid monomers used in the PA synthesis are given in Table 4. Fatty acid-based PAs are semicrystalline in nature and are soluble in common chlorinated organic solvents. These polymers display low melting temperatures, usually in the range of 20–90 °C.

**Table 4:**
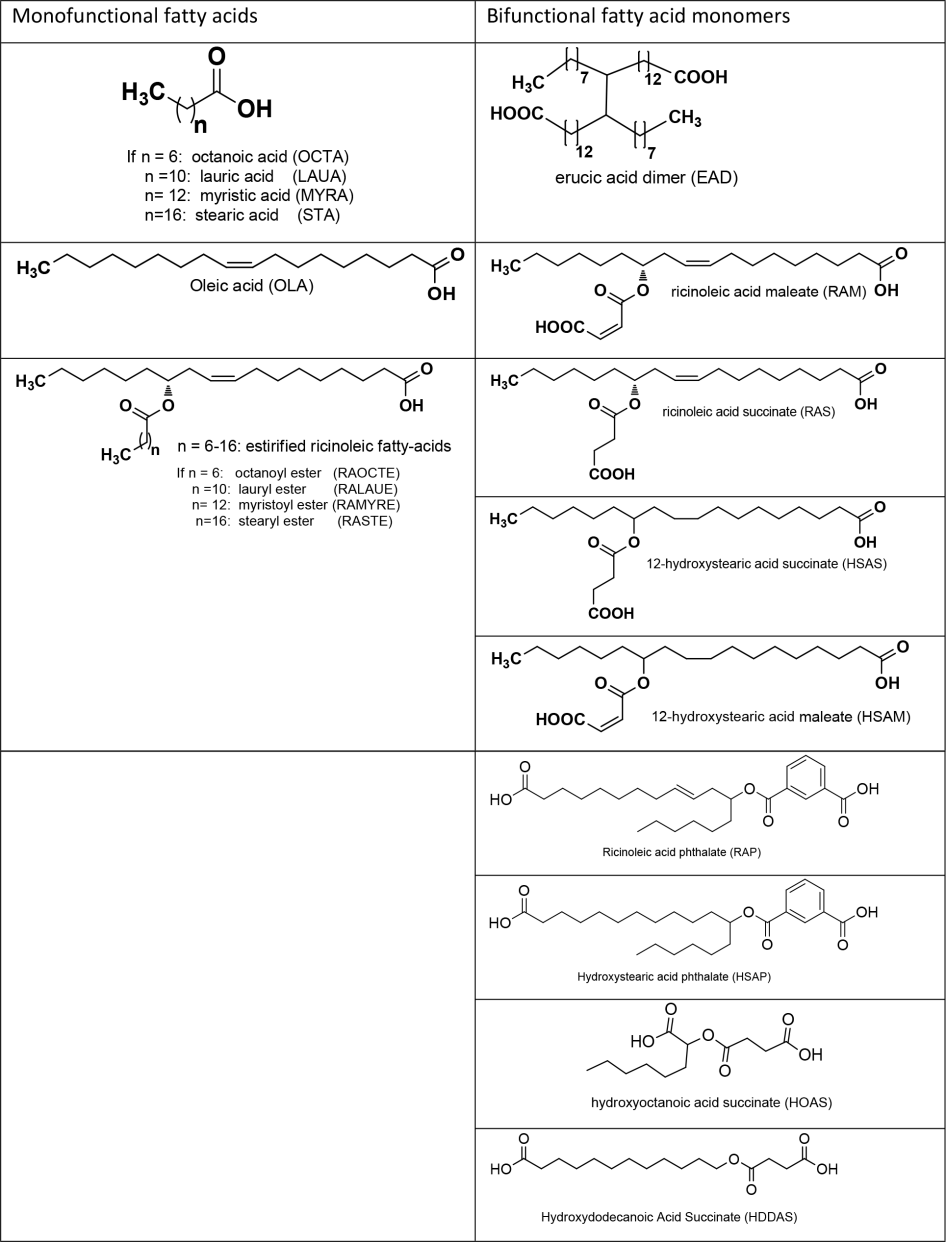
Chemical Structures of Mono and Bifunctional Fatty Acid Monomers Used for PA Synthesis

#### 2.6.1 Polyanhydrides with Fatty-Acid End Chains

**Scheme 5:**
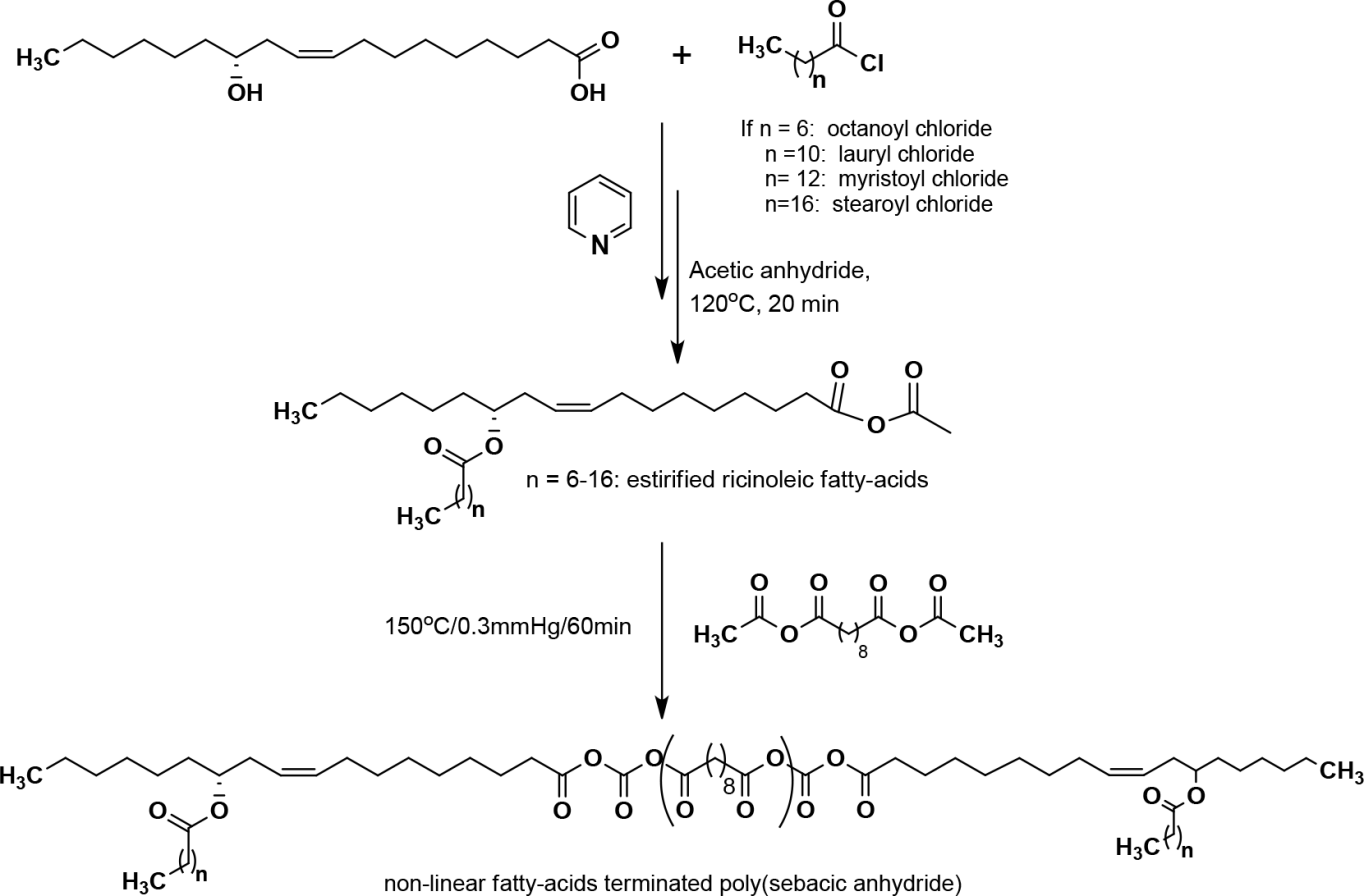
Synthetic Route for Nonlinear Fatty-Acids Terminated Poly(sebacic anhydrides) Reproduced with permission from ref (106). Copyright 2001 American Chemical Society.

To improve the hydrophobicity and control the degradation rate of the aliphatic PAs, our group reported a series of fatty acid terminated poly(SA).^105^ These polymers were prepared by melt condensation between the acetate anhydrides of various saturated linear fatty acids with different carbon chain lengths, C8–C18 (octanoic acid, lauric acid, myristic acid, and stearic acid), and sebacic anhydride oligomers. Incorporation of high fatty acid loading (10% to 30% w/w) leads to low molecular weight fatty-acid terminated poly(SA). The polymers showed a slow hydrolytic degradation profile compared to poly(SA) due to the increasing hydrophobic content of terminal fatty acids that prevent water from penetrating into the polymer matrix to cleave the labile anhydride bond. This affects the drug release profile. Esterified ricinoleic acid with aliphatic fatty-acid of C8–C18 chain lengths have been used as P(SA) terminators (Scheme 5).^106^ Incorporation of nonlinear fatty-acid chain terminators into the poly(SA) leads to improved hydrophobicity and a decrease in polymer crystallinity as compared to the poly(SA). Nonlinear fatty acid terminated poly(SA) are hydrolytically degraded into their constituents over a period of weeks, which allows for the constant release of the drug methotrexate from the polymer matrix.

#### 2.6.2 Polyanhydrides Based on Nonlinear Fatty-Acid Dimers

Dimers of erucic acid (EAD) have been used for the synthesis of fatty acid-based PAs. The homopolymer obtained from this monomer is highly viscous. However, its copolymerization with increasing amounts of SA (10–90%) leads to the formation of solid polymers with melting points ranging from 30 to 85 °C.^107^ These polymers have been investigated as drug delivery carriers of carboplatin, methotrexate, tetracycline, gentamicin, paclitaxel, heparin, and bupivacaine.^107−109^ For example, disk-shaped implants of poly(EAD-SA, 50:50 w/w) showed 90% bupivacaine release in 35 days, 100% of cefazolin sodium released in 14 days, but it was noted that only 15% of paclitaxel was released over a period of 77 days.^108^ Fatty-dicarboxylic acid monomers made from the esterification of ricinoleic acid (RAM, RAS, HSAS, and HSAM) have been used for the synthesis of fully degradable PAs (Table 4).^110,111^ A series of homopolymers and copolymers with SA were synthesized through melt-polymerization. The polymers showed good film formation ability with excellent tensile strength. The hydrolytic degradation of polymers into natural compounds and their sustained release of ciprofloxacin and methotrexate have been studied *in vitro*. These polymers are biocompatible and degrade under *in vivo* conditions within 2 months.

**Figure 3:**
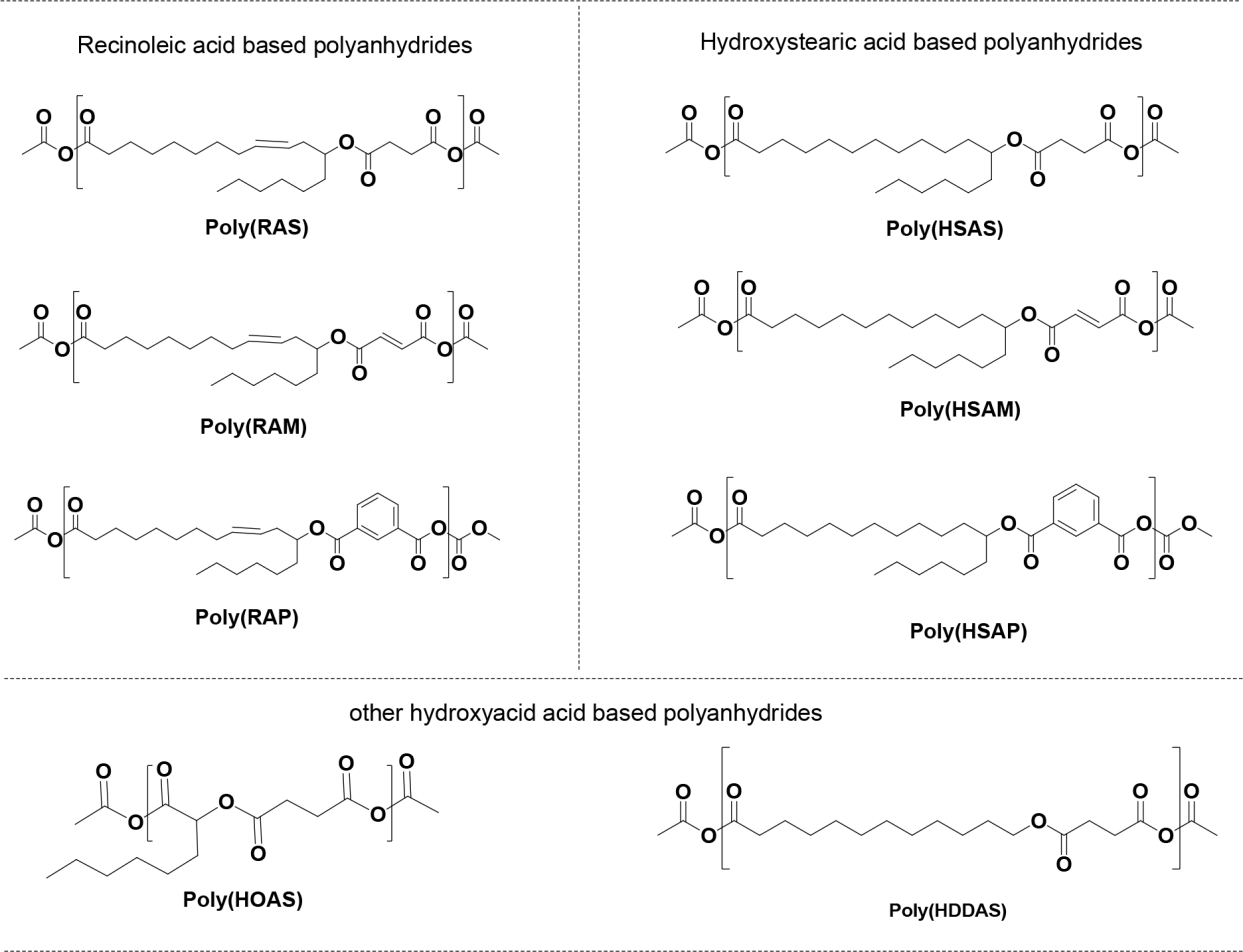
Chemical structures of various poly(ester-anhydrides) synthesized through melt-condensation polymerization of activated ester diacid monomers.

Recently, our group studied the stability and hydrolytic degradation properties of a series of pasty poly(ester-anhydride)s such as ploy(RAS), poly(RAM), poly(RAP), poly(HSAS), poly(HSAM), poly(HSAP), poly(HOAS), and poly(HDDAS) based on the effect of ester bonds, hydrophobic side chains, phenyl moieties, and their distance from anhydride bonds.^54^ These polymers are obtained from melt condensation polymerization of the corresponding bifunctional fatty acid monomers RAS, RAM, RAP, HSAS, HSAM, HSAP, HOAS, and HDDAS (Table 4 and Figure 3).The bifunctional fatty acid monomers are synthesized by the esterification reaction between ricinoleic or other hydroxy acids and cyclic anhydrides such as succinic, maleic, and phthalic anhydrides. The polymers were stable at room temperature for 3 months under an inert atmosphere as revealed by the stability studies. Due to the insertion of hydrophobic side chains or aromatic units adjacent to the anhydride bond in the poly(ester-anhydride)s slower their degradation rate. This is due to the aliphatic chain or aromatic units masking the anhydride bond alternatively in the polymer structure, thus controlling the hydrolytic degradation of the anhydride bond in the polymer. These phenomena lead to stable polymers, which can be easily handled at room temperature for the controlled delivery of drugs under normal conditions. The PAs thus designed are low melting solids and are pasty in nature. The applicability of these polymers is reported for their *in vitro* drug release pattern using ibuprofen. Compared to the other poly(ester-anhydrides), the aromatic polymers poly(RAP) and poly(HSAP) displayed sustained release of ibuprofen at 50% and 40% over a period of 28 days.

#### 2.6.3 Insertion of Unsaturated Fatty Acids into the Polyanhydride Backbone

**Scheme 6:**
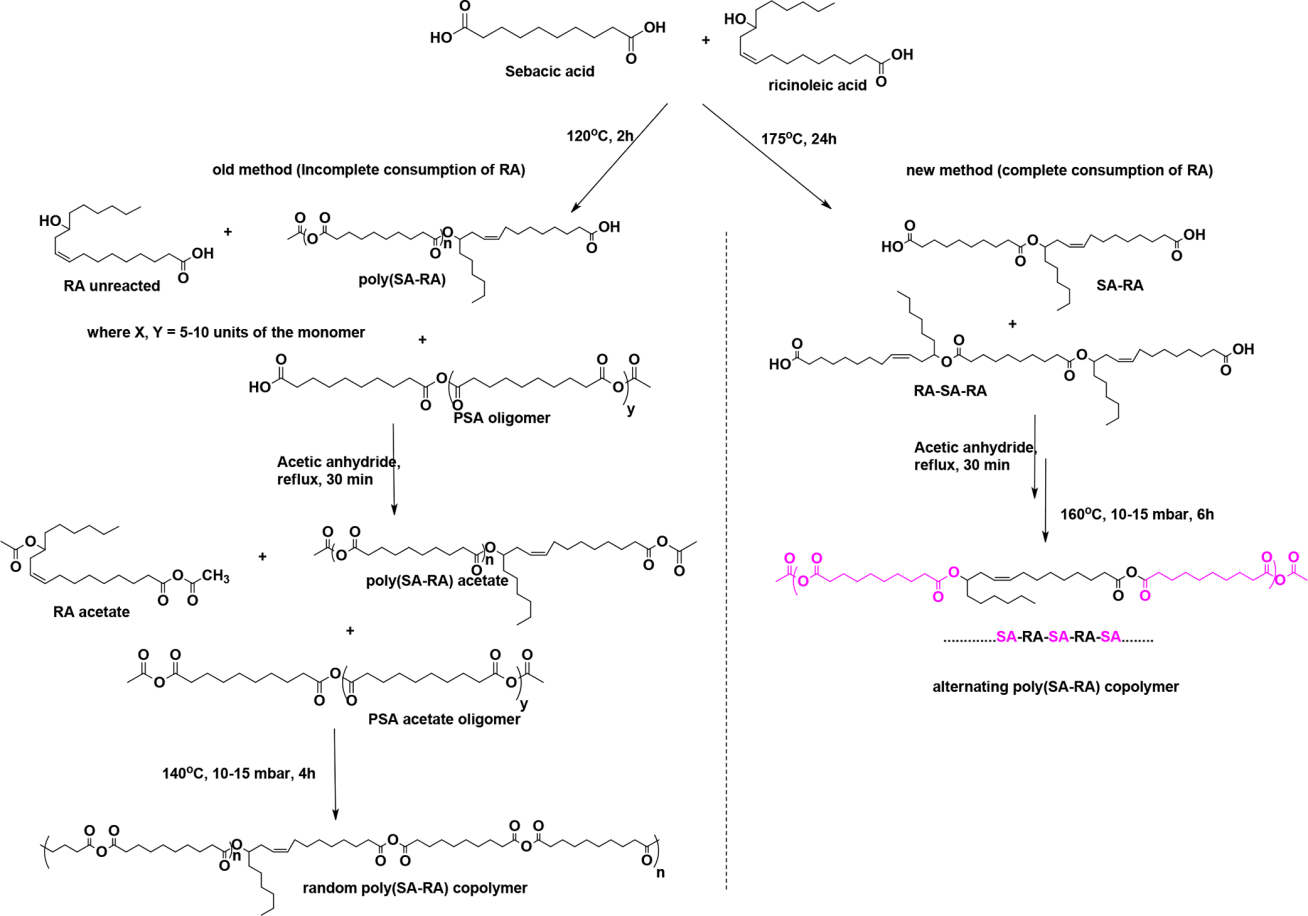
Difference between the Synthetic Routes for Random and Alternating Poly(SA-RA) Reprinted from Stable Polyanhydride Synthesized from Sebacic acid and Ricinoleicacid, Vol. 257, Zada, M. H.; Basu, A.; Hagigit, T.; Schlinger, R.; Grishko, M.; Kraminsky, A.; Hanuka, E.; Domb, A. J., pp. 156–162, Copyright 2017, with permission from Elsevier.

Random incorporation of ricinoleic acid (RA) into poly(SA) chain resulted in pasty polymers (Scheme 6).^42^ The hydroxyl group of RA reacts with the anhydride bond along poly(SA), leading to transesterification and formation of oligomers with carboxylic acid terminals. Melt condensation of these oligomers in acetic anhydride leads to the generation of random poly(SA-RA) copolymers. These polymers are pasty and freely injectable at 37 °C. The increase of RA content in the polymer leads to a decrease in the melting point and crystallinity.

With an aim to synthesize stable poly(ester-anhydrides), a modified protocol used for the synthesis of alternating poly(SA-RA) (3:7), which is stable at the room temperature, was employed (Scheme 6).^43,44^ Using this method, dimers and trimers of RA-SA or RA-SA-RA have been synthesized and polymerized into PAs with alternating SA-RA structure. The regular occurrence of the RA side chain in the polymer causes steric hindrance of the RA side chain on each anhydride bond along the polymer chain, which reduces anhydride interchange and hydrolysis. The alternative poly(SA-RA) polymers are stable at room temperature for more than 2 years.^45,46^ The drug-releasing efficacy of the random poly(SA-RA) depends on the percentage of RA content. An increase in RA content increases the hydrophobicity of the polymer resulting in more sustained drug release. Random/alternative poly(SA-RA) have been used for the sustained release of paclitaxel,^47^ gentamycin,^44,48^ and dexamethasone.^47^ Different fatty acid-based PAs used for the drug delivery applications are given in Table 5.

**Table 5:** Various Fatty-Acid Based PAs Developed for Drug Delivery

PAs with fatty-acid end chains				
name of the polymer	*T*_m_ [°C]	Δ*H* [J/g]	application	ref
Poly(OCTA-SA) (10:90 and 30:70)	70–73	73–81	Sustained release of methotrexate and bupivacaine free base	(105)
Poly(LAUA-SA) (10:90 and 30:70)	70–71	77–94		
Poly(MYRA-SA) (10:90 and 30:70)	75–78	79–83		
Poly(OLA-SA) (10:90 and 30:70)	73–74	60–69		
Poly(STA-SA) with different compositions	71–78	97–104		
Poly(RASTE-SA) (3:7)	79	65	Sustained release of methotrexate	(106)
Poly(RAMYRE-SA) (3:7)	78	68		
Poly(RALAUE-SA) (3:7)	78	79		
Poly(RAOCTE-SA) (3:7)	77	67		
PAs based on nonlinear fatty acid dimers				
Poly(EAD-SA) with different compositions of SA from 10 to 90%	35–82	-	Sustained release of range drugs such as methotrexate, tetracycline, gentamicin, bupivacaine free base, cefazolin, taxol, and heparin	(107−109)
Poly(RAM)	Viscous oil	-	Invitro release of ibuprofen	(54, 110, 111)
Poly(RAS)	Viscous oil	-		
Poly(HSAS)	Semisolid	-		
Poly(HSAM)		-		
Poly(RAM-SA) with different composition of RAM from 10 to 50%	66–86	65–119	Sustain release of the drugs ciprofloxacin and methotrexate	
Poly(RAS-SA) (50:50)	61	66		
Poly(HSAS-SA) (50:50)	70	78		
Poly(HSAM-SA) (50:50)	67	51		
Insertion of unsaturated fatty acids into the PA backbone				
Random poly(SA-RA) with different composition of SA from 10 to 80%	25–78	12–133	Release of anticancer cisplatin, paclitaxel, gentamycin and dexamethasone	(42−44, 47−49)
Alternative poly(SA-RA) (70:30)	36	-		

**Scheme 7:**
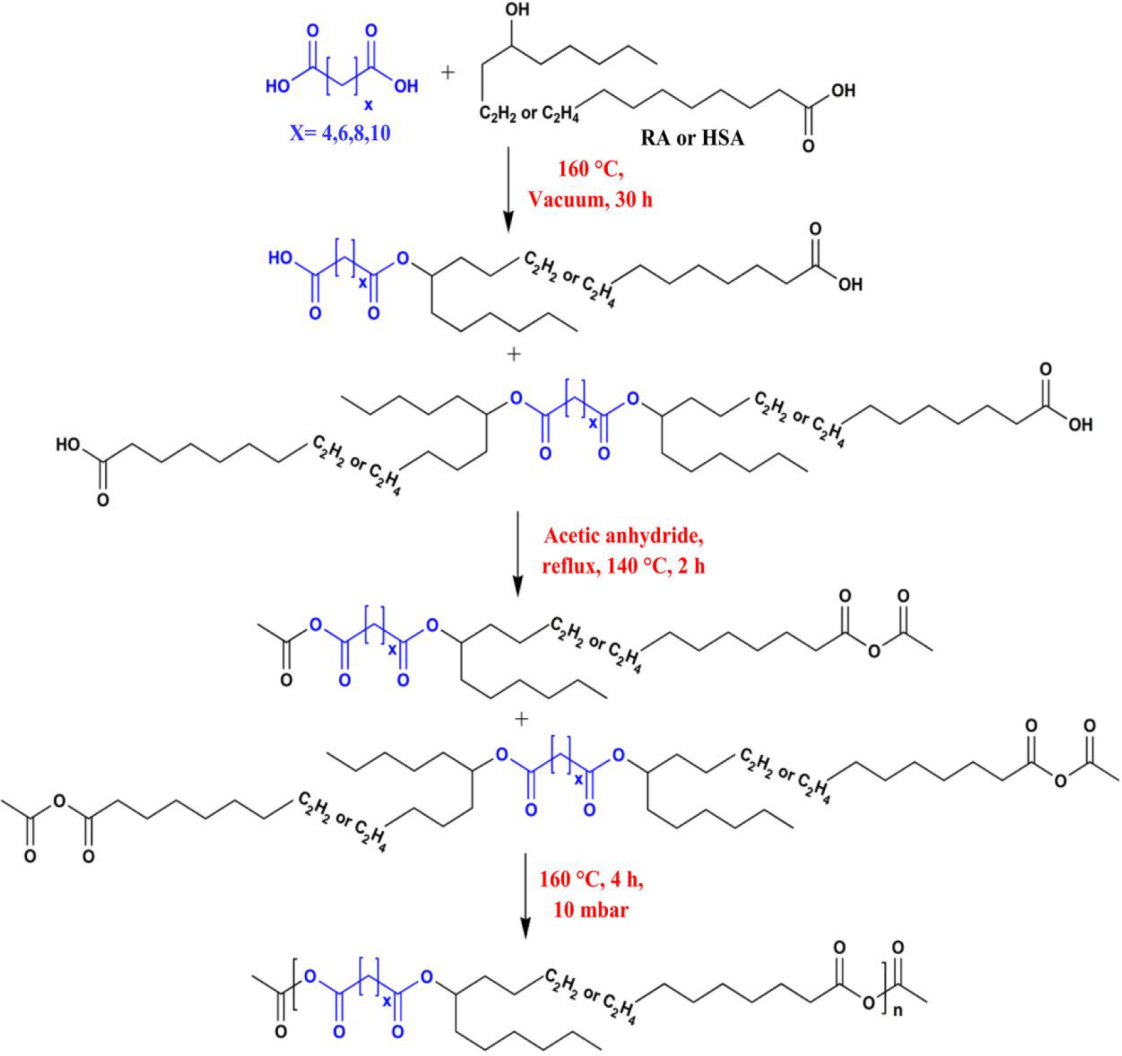
General Synthesis Protocol for the Synthesis of Poly(ester-anhydrides) from Dicarboxylic Acids (AA/SA/SUA/DA) and Hydroxyalkanoic Acids (RA/HSA) Reprinted with permission from ref (49), Copyright 2022 John Wiley & Sons Ltd.

PAs with controlled molecular weights have been reported (Scheme 7).^49^ Here, the ester diacid monomer was synthesized by the stepwise addition of the hydroxy alkanoic acid molecules into melted dicarboxylic acids. The ester monomer is then melt-polymerized in the presence of a molar equivalent amount of acetic anhydride into stable poly(ester-anhydrides). The advantage of this synthetic route is that it allows for the complete consumption of hydroxy acids without any self-condensation. Moreover, the polymers obtained by this method are very stable with controlled and reproducible molecular weights.

## 3 Synthetic Methods

**Scheme 8:**
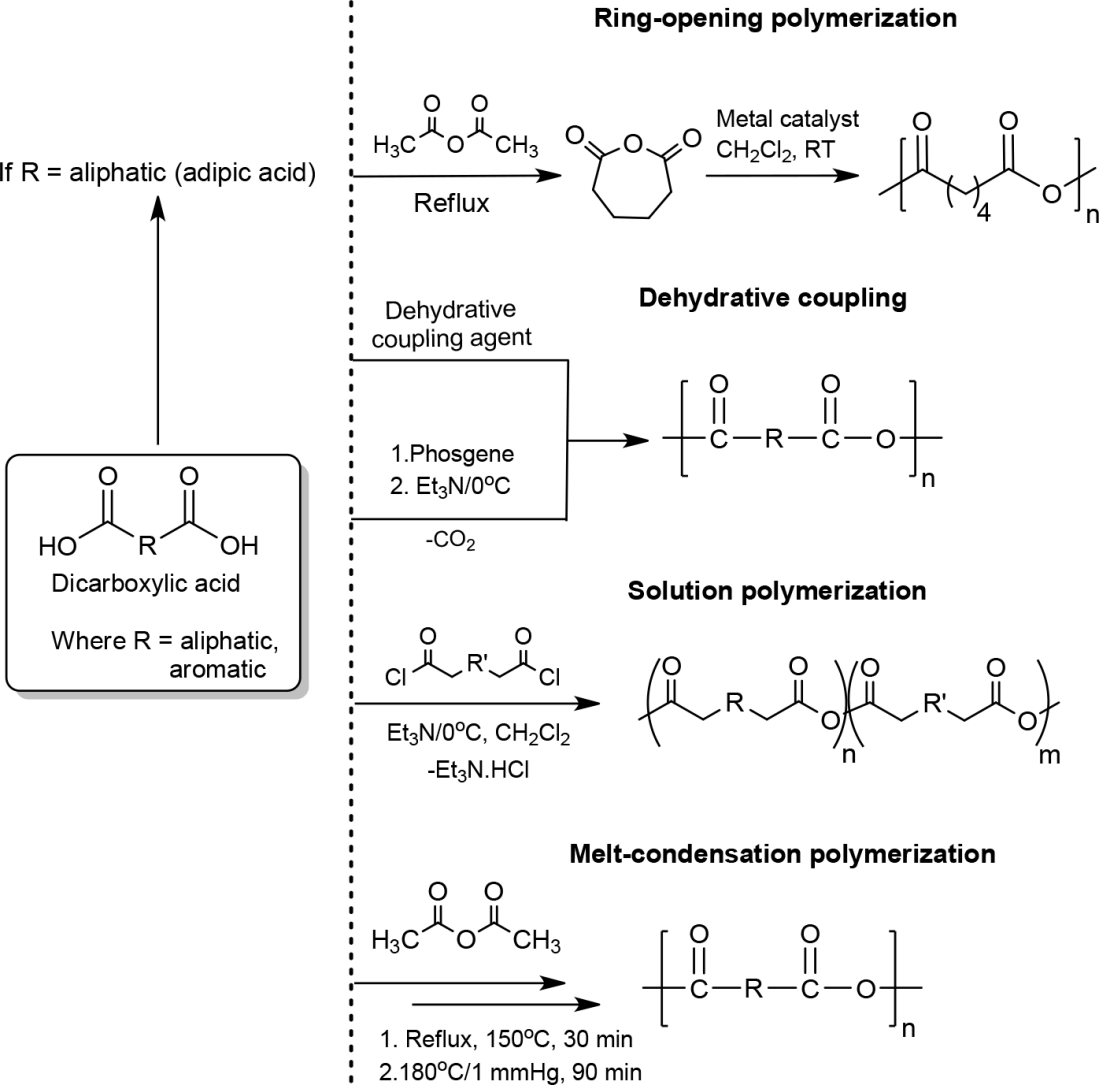
General Synthetic Methods for PAs

PAs has been synthesized by melt condensation, solution polymerization, dehydrative coupling, and ring-opening polymerization (Scheme 8).^10−14,19−23^ Among various synthetic methods developed, the “melt condensation” received significant importance due to the straightforward synthesis from dicarboxylic acid monomers.

### 3.1 Melt Condensation Polymerization

Melt condensation takes place in two stages: (1) Formation of acetyl-terminated anhydride prepolymers with a degree of polymerization 1–20. This can be obtained by the reaction of dicarboxylic monomers with excess acetic anhydride. (2) Involving polymerization of prepolymer units at elevated temperature under high vacuum conditions to get a PA with a degree of polymerization of 100 to over 1000. This method is successful in the synthesis of a variety of aliphatic, aromatic, aliphatic-aromatic, and fatty acid-based Pas.^10−14,19−23,34−38^ A variety of coordination metal catalysts has been used for the synthesis of high molecular weight PAs with shorter reaction time. Some of these catalysts include calcium oxide, barium oxide, cadmium acetate, diethyl zinc, and calcium carbonate. Except for calcium carbonate, the use of other metal catalysts for the potential synthesis of medicinal biodegradable PAs is limited because of their toxicity.

### 3.2 Solution Polymerization

The polymerization was carried out per the Schotten-Baumann method. The reaction was conducted by the dropwise addition of diacid chloride into an ice-cooled solution of a dicarboxylic acid monomer under inert atmospheric conditions. The reaction is facilitated by the addition of acid acceptors such as triethylamine, pyridine, etc. The polymerization takes place instantly upon contact with the monomers and is completed within a shorter reaction time period of 1h. The solvent used for this reaction can be a mixed solvent or a single solvent like dichloromethane, chloroform, benzene, and ethyl-ether. The formation of high molecular weight polymers depends on the order of addition of the monomer. For example, the addition of diacid solution to the diacid chloride constantly produces high molecular weight polymers.^10−14,19−23^ The advantage of this method is a synthesis of PAs from heat-sensitive diacid monomers such as dipeptides and other therapeutically active agents under ambient reaction conditions.

### 3.3 Dehydrative Coupling

The method of dehydrative coupling of a diacid monomer was investigated.^63^ PAs were synthesized from a dicarboxylic acid monomer using a dehydrative coupling agent at room temperature. The most effective coupling agents reported are bis[2-oxo-3-oxazolidinyl]phosphinic chloride and *N*-phenylphosphoroamidochloridate.^63^ Other coupling agents such as dicyclohexylcarbodiimide, chlorosulfonyl isocyanate, and 1,4-phenylene diisocyanate were also studied but they yielded only oligomers. Phosgene and triphosgene were also used for the synthesis of PA.^112^ When using such coupling agents, the acid scavenger was isolated by using insoluble acid acceptors such as poly(4-phenylpyridine)(PVP) or triethyl amine (TEA) or K_2_CO_3_.^112^

### 3.4 Ring Opening Polymerization (ROP)

ROP is a well-known and frequently applied method for the synthesis of polymers from the heterocyclic monomers.^4^ Examples of polymers obtained from the ROP include polyesters, polycarbonates, polyphosphazene, and polypeptides. In the ROP reaction, the reactive center in the terminal end of the polymer chain added to another cyclic monomer such a way growth of the polymer chain occurs. In this context of the research focus, ROP used as an alternative approach to the synthesis of biodegradable PAs from the cyclic anhydride monomers. For example, Albertsson and Lundmark and co-workers investigated for the synthesis of polyadipic anhydride from cyclic adipic anhydride (oxepane-2,7-dione) using different types of cationic (AlCl_3_, BF_3_.(C_2_H_5_)_2_O), anionic (CH_3_COOK, NaH), coordination (stannous-2-ethylhexanoate, dibutyl tin oxide)and metal (1% ZnCl_2_) catalysts.^113,114^ Synthesis of PA through ROP involves in two steps: 1) synthesis of cyclic anhydride monomer from the dicarboxylic acid unit, and 2) Polymerization of the cyclic monomer in the presence of a catalyst. The cyclic monomer was usually prepared by the heating of diacid derivatives in the presence of acetic anhydride under reflux conditions. Polymerization occurred through the addition of cyclic monomer into the active species of the catalyst. This addition proceeds through cleavage of the acyl-oxygen bond of the cyclic anhydride.

### 3.5 Radical Polymerization

In the radical-mediated pathway, the methacrylated anhydride monomers were developed for the synthesis of biodegradable Pas.^100,115^ Methacrylated anhydride monomers provide an opportunity to conduct radical-mediated polymerization.^10−14^ This would be initiated by using a variety of photo or thermal or redox initiators. Photopolymerization has significant advantages as it allows spatial and temporal control.^10−14^ Moreover, the photopolymerization also occurred often quite rapidly. Examples of commonly used photo initiators for the synthesis of PA from methacrylate anhydride monomers are camphorquinone (CQ) and 2,2-dimethoxy-2-phenylacetophenone. In this context, the method for the synthesis of cross-linked PAs and oligomers from methacrylate anhydride monomers through photopolymerization process was first reported by the Langer group.^100,115^ The monomers mCPP, mCPH, and mSA were synthesized by reacting diacid molecules SA, CPP, and CPH with methacrylic anhydride under heating conditions. These monomers were unstable, as they undergo anhydride exchange (disproportionation) to form oligomer units.^115^ However, this behavior does not affect the polymerization process. The photopolymerization of these dimethacrylated anhydride monomers leads to biodegradable cross-linked PAs (poly mSA, poly mCPH, and poly mCPP). The applicability of these cross-linked PAs with controlled degradation profile has shown excellent histocompatibility *in vivo.*

**Scheme 9:**
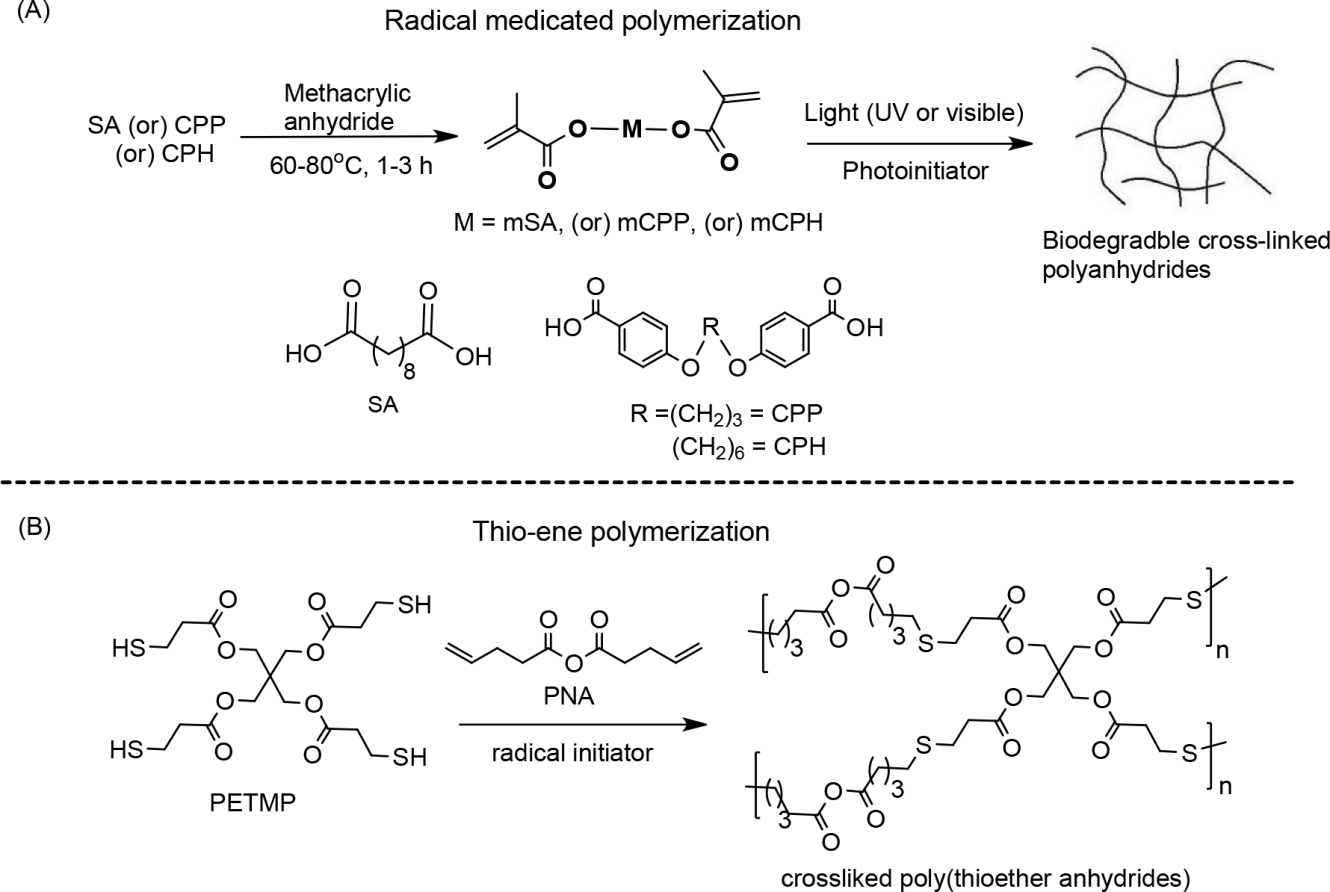
Synthesis of Cross-Linked PAs: (a) Synthetic Scheme for Dimethacrylated Anhydride Monomers and Their Radical-Mediated Photopolymerization; (b) Synthesis of Thioether Based Cross-Linked PA Based on Thio-ene Photopolymerization of PETMP and PNA Reprinted by permission from Springer Nature Customer Service Centre GmbH: Springer Nature Nature Biotechnology Photopolymerizable Degradable Polyanhydrides With Osteocompatibility, Anseth, K. S.; Shastri, V. R.; Langer, R. Copyright 1999. Used with permission of Royal Society of Chemistry, from Elastomeric and Degradable Polyanhydride Network Polymers by Step-growth Thiol–ene Photopolymerization, Shipp, D. A.; Mc Quinn, C. W.; Rutherglen, B. G.; Mc Bath, R. A., 2009; permission conveyed through Copyright Clearance Center, Inc.

Another method reported for the synthesis of PA based on the radical mediated pathway is thio-ene “click” polymerization. The method is also called alkene hydrothiolation. Per this method, the anhydride containing monomers such as dithiols and dialkenes react to each other in the presence of radical initiator or catalyst and form a thioether based cross-linked PAs. The polymerization can be performed under ambient conditions without any organic solvent. The polymerization involves a step-growth mechanism that can lead to more uniform cross-link densities with high conversions in shorter timeframes. An example of the preparation of cross-linked PAs was demonstrated by the photopolymerization of 4-pentenoic anhydride (PNA) and pentaerythritol tetrakis(3-mercaptopropionate) (PETMP) in the presence of 0.1 wt % of 1-hydroxycyclohexyl phenyl ketone initiator under UV-light exposure is shown in Scheme 9.^87^ However, using this strategy for the synthesis of biodegradable medical PAs is rare.^90,116^

**Scheme 10:**
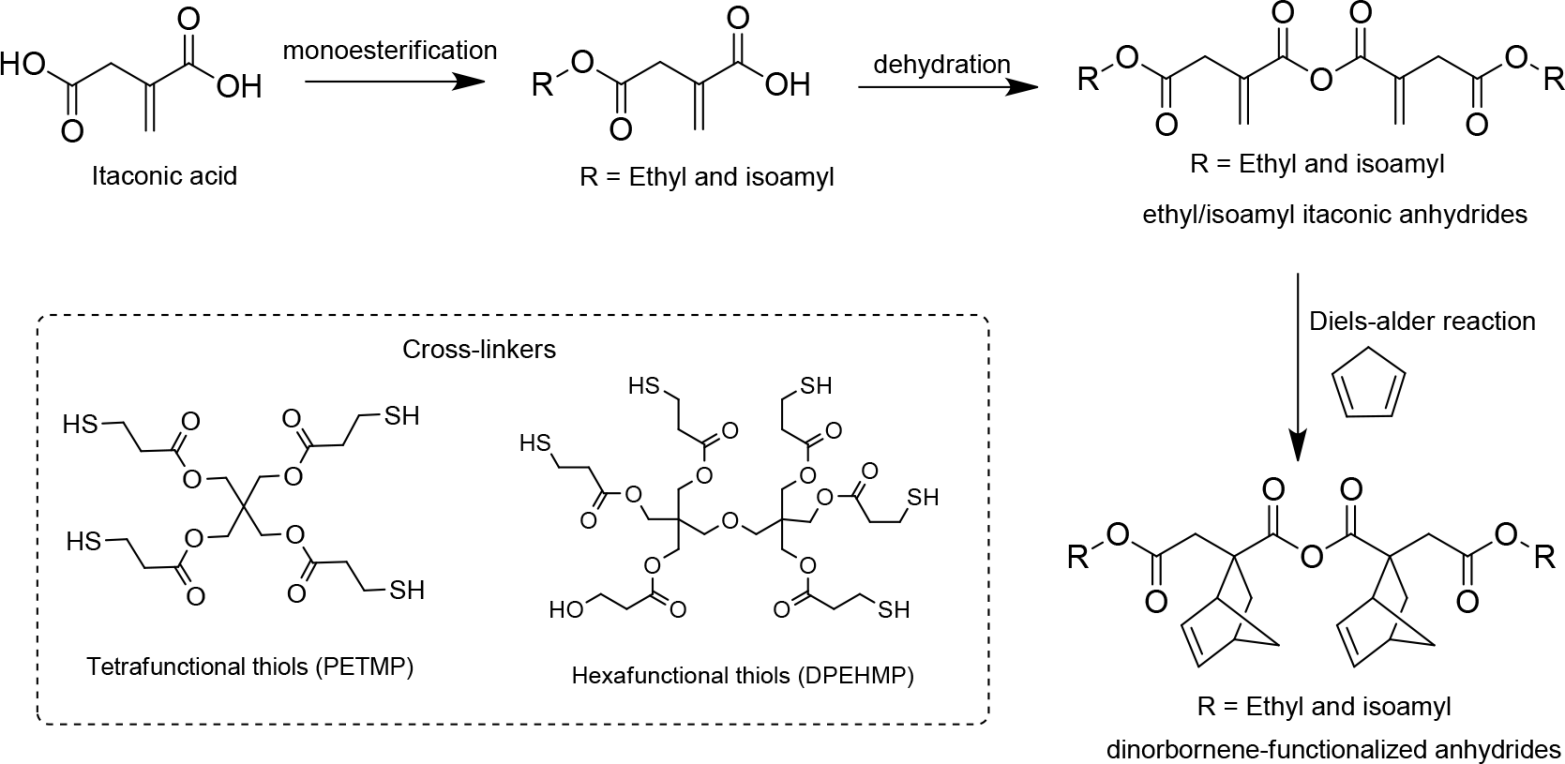
Synthetic Route for Itaconic Anhydrides and the Chemical Structures of Tetrafunctional Thiols (PETMP)/ Hexafunctional Thiols (DPEHMP) Used for the Synthesis of Cross-Linked PA-Networks through Thio-enechemistry Used with permission of Royal Society of Chemistry, from Degradable Polyanhydride Networks Derived from Itaconic Acid, Sajjad, H.; Lillie, L. M.; Lau, C. M.; Ellison, C. J.; Tolman, W. B.; Reineke, T. M., Vol. 12, 2021; permission conveyed through Copyright Clearance Center, Inc.

Recently, Sajjad et al. reported the use of itaconic acid as a valuable bioderived feedstock to create a degradable cross-linked PA networks through thio-ene photopolymerization.^117^ The starting monomers of itaconic anhydrides, such as ethyl itaconic anhydride and isoamyl itaconic anhydride, were obtained from esterification of itaconic acid with ethanol/isoamyl alcohol lead to itaconic monoesters, which followed by the self-condensation with acetic anhydride (Scheme 10). Further, the cycloaddition of ethyl/isoamyl itaconic anhydride with cyclopentadiene leading to other norbornene-functionalized anhydrides. The thio-ene polymerization of all these itaconic anhydrides with tetrafunctional thiols/hexafunctional thiols affords a series of cross-linked PA networks. These polymers are susceptible to maintaining the degradation properties in the PBS and normal seawater over a period of 60 days at 50 °C. In the basic environments, these polymers showed faster degradation behavior. The cross-linked PA network made-up of the hexa-thiol cross-linker possesses higher stiffness and *T*_g_ than the polymer constructed with tetra-thiol due to high cross-linking density.

## 4 Hydrolytic Degradation of Polyanhydrides

Biodegradation of medical polymers is a process that designates the cleavage of polymer materials into small fragments of oligomers and monomers due to hydrolysis or enzymatic action.^1^ In this context, the hydrolytic degradation of many PA-based biomedical implants have been studied irrespective of its geometry such as slab, cylinder, matrix, or microspheres, etc.^10−14,19−23^

PAs are hydrolytically unstable due to the anhydride linkage. The rate of hydrolytic degradation for PAs is faster compared to other classes of biodegradable polymers such as polyesters, polyorthoesters, polyamides, etc.^13,14^ These polymers can compose diacid-therapeutics; therefore, the chance of the inflammatory response from degraded products of the polymer can be minimized with improved biocompatibility.^14,118^ These features allowed the use of PAs as drug-release carriers. In the drug delivery process, the drug is loaded within the polymer matrix, and it delivers in a controlled manner at the targeted specific site over the course of polymer degradation. The rate of erosion is controlled by varying the polymer composition, type of monomer (hydrophilic or hydrophobic monomers), etc.

**Scheme 11:**
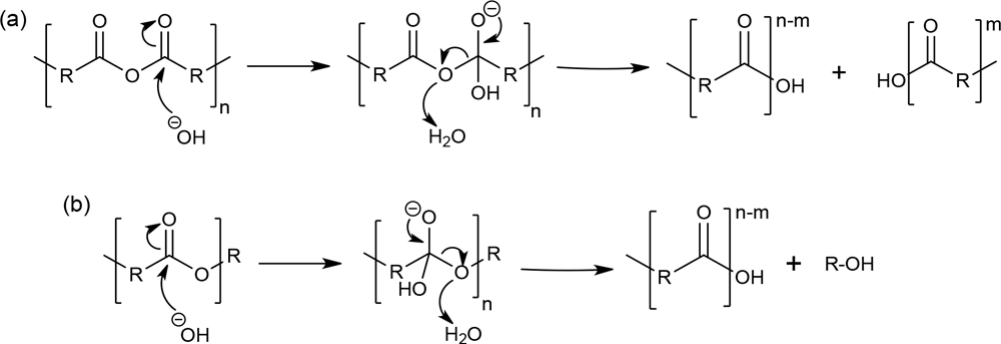
Hydrolysis of (a) Polyanhydrides and (b) Polyesters Reproduced with permission from ref (20), Copyright 2012 Elsevier. Reprinted from *Biodegradation of Polymers*, Vol. 9, Murthy, N.; Wilson, S.; Sy, J. C., pp. 547-560, Copyright 2012, with permission from Elsevier.

PAs predominantly undergo base catalyzed hydrolysis for degradation.^20^ The degradation mechanism of PAs is like that of polyesters. Initially, the hydroxyl group is added to the carbonyl carbon of the anhydride bond, which results in the formation of a tetrahedral intermediate. Like polyesters, this tetrahedral intermediate does not regenerate the starting anhydride through the expulsion of the hydroxide anion. This is due to the low p*K*_a_ when leaving carboxyl group. The tetrahedral intermediate is unstable as it is undergoing hydrolysis in the presence of water resulting in the cleavage of the attached ester into a carboxylic acid. The base-catalyzed degradation mechanism for the PAs and polyesters is shown in Scheme 11.

## 5 Factors Influencing the Degradation of Polyanhydrides

The rate of the polymer degradation is influenced by several factors, including. pH of the surrounding medium, the biological environment, hydrophilicity and crystallinity of the PA, and type of the drug loading in the PA matrix.^11^

### 5.1 pH

PAs undergo base catalyzed hydrolysis, therefore the pH of the surrounding medium significantly influences the degradation rate of the PA matrices.^119^ The erosion rate of the poly(FAD-SA) copolymer at different pH (pH 1–9) was reported. Poly(FAD-SA) showed 1.3–2.0-times faster degradation at pH = 9 than the degradation rate that was observed at pH = 7 and this in turn is 8–10-times faster than the degradation at pH = 1–5.

### 5.2 Hydrophilicity

To study the effect of monomer hydrophilicity in polymer degradation kinetics,^64^ a series of aliphatic PAs based on varying aliphatic monomer carbon chain length from 4 to 12 was used. The solubility of these aliphatic diacid monomers in water decreased when increasing carbon chain length. The rate of degradation of the aliphatic PAs is directly proportional to the water solubility of their respective monomers. For example, PAs prepared from the diacid monomers with 7–10 carbon chain lengths showed 20% weight loss in 48 h, compared to the 70% weight loss noted in polymers prepared with the shorter diacid chain lengths of 4–6. The erosion of poly(CPP-SA) copolymers was regulated by changing the molar compositions of the starting hydrophobic CPP and hydrophilic SA monomers.^58^ The erosion was studied by incubating poly(CPP-SA) circular discs in PBS (pH = 7.4) at 37 °C for a period of 14 weeks. The increase of hydrophilic SA content in poly(CPP-SA) increases the erosion rate. In fact, the inclusion of high hydrophilic SA in the former copolymer displayed an 800-fold increases erosion rate compared to homo poly(CPP). Using this strategy, one can construct PA-based drug-loaded matrices with the controlled drug-release properties that ranged from over a year to a few months.

### 5.3 Crystallinity

Water diffusion into the surface layers of the polymer matrix is dependent on the crystalline nature of the polymer. In contrast, amorphous structures showed a high degradation rate due to the irregular arrangement of the polymer structure.^120^ A series of cyclo aliphatic PAs, i.e., poly(cis CHDA-*co*-trans CHDA), poly(CHDA-AD) with different chemical compositions starting from cis/trans-1,4-cyclohexanedicarboxylic acid (CHDA), and adipic acid (AD) were degraded in pH = 7.4 PBS solution at 37 °C. The degradation studies of poly(cis CHDA-*co*-trans CHDA) with different cis:trans compositions, revealed that the cis-CHDA units in the copolymer structure were degraded faster compared to trans-CHDA components. This is because high crystalline trans-CHDA showed poor water penetration into the matrix, thus leading to a decreased degradation rate of the polymer compared to the polymers containing cis-CHDA components. However, the copolymers poly(CHDA-AD) with different CHDA:AD compositions (20:80; 40:60; 80:20) showed higher crystallinity than poly(CHDA) (cis:trans composition = 40:60), but the degradation rate of the former is higher than the latter. This is due to the inclusion of aliphatic AD in the copolymers which increases the hydrophilicity and exerts a stronger effect than crystallinity, thereby leading to faster degradation which can be seen in the case of poly(CHDA-AD) copolymers as compared to poly(CHDA).

The blending approach controls the degree of crystallinity of the polymer. The mixing of two polymers lowers their degree of crystallinity, which yields high degradation kinetics. For example, the blend of poly(adipic anhydride) with poly(trimethylene carbonate) showed a faster mass loss than pure poly(adipic anhydride).^121^ Recently, Lienkamp et al. reported poly(SA)/poly(AA) blends, which displayed an accelerated degradation rate at their initial phase compared to the poly(SA).^122^ copolymerization is another approach to control the degree of crystallinity of the polymer. For example, the copolymer films of poly(salicylic acid-co-sebacic acid) showed faster degradation kinetics than the poly(sebacic anhydride) and released the antimicrobial salicylic acid due to low crystallinity.^122^

### 5.4 Effect of Drug Loading

The physical properties of the encapsulated drug affect the erosion rate of the PA matrix. Tablets loaded with mannitol, insulin, and stearic acid showed different degradation rates, with mannitol and insulin tablets degrading at ∼6 wt % day, while stearic acid degrades at ∼2 wt % day.^119^ In another example, ibuprofen or pseudophedrine hydrochloride incorporated poly(CHDA) wafers, showed faster release of pseudophedrine hydrochloride from the polymer matrix than ibuprofen.^120^ The hydrophilic nature of pseudophedrine hydrochloride diffuses more easily into the polymer matrix, thereby allowing water to occupy the empty spaces in the matrix. This leads to enhanced degradation and faster release kinetics of pseudophedrine hydrochloride compared to ibuprofen.

### 5.5 Other Factors

The degradation of the PA is also influenced by the water diffusion rate, polymer hydrolysis rate, matrix dimensions, as well as sample thickness. The polymer degradation takes place either through bulk or surface erosion. Göpferich et al. calculated the critical device dimension (L_critical_) based on the mode of erosion number (ε).^123^ Per the calculation, if the sample matrix size is larger than L_critical_, then it will undergo surface erosion. Perhaps, if the device dimensions are lower than the L_critical_, bulk erosion is dominant. The critical length for the PAs is 75 μm. Karen Lienkamp et al. recently demonstrated the thickness effect on the degradation rate of the polymer. Based on their findings, the higher thickness PA showed a slow degradation profile compared to low thickness PAs. For example, free-standing poly(SA) pellets with 190–670 μm have shown a slower degradation rate than compared poly(SA) pellets with a thickness of <1 μm.^124^

## 6 Applications

The development of biodegradable polymers for the controlled release of drugs, vaccines, and other therapeutics is a major research area in the biomedical field. In this direction, the PAs were using as biocompatible drug delivery carriers for a long time. The significant characteristics of PAs such as controlled surface erosion and zero-order release kinetics have made them desirable as therapeutic delivery vehicles. In this section, we shall outline the use of novel PAs in the biomedical field as medical devices and vaccine or drug or peptide delivery vehicles with a few recent examples.

### 6.1 Use of Polyanhydrides in Medical Electronic Devices

Due to the rapid hydrolytic degradability of PAs that have also been utilized to construct a few high-end transient medical electronic devices. For example, a biodegradable primary battery as a potential power source for temporary biomedical implants has been reported.^125^ This battery was constructed based on the biodegradable metal foils and PAs package. They have used magnesium foils as an anode, and the metal foils based on the Fe, W, or Mo that serve to the function of a cathode. The PA shell is prepared from the mixing of UV-curable monomers such as pentaerythritol tetrakis (3-mercaptopropionate), 4-pentenoic anhydride, and poly(ethylene glycol) diacrylate (molar ratio of 5:7:3) in the presence of photo initiator “2,2-dimethoxy-2-phenylacetophenone (0.4 wt %)” under UV-light (6 mW cm^–2^) for 10 min. The entire electrode package was encapsulated within the PA shell, and the resultant battery is biodegradable as it degrades naturally inside the body.

In another study, a bioresorbable silicon-based electronic brain sensor that was encapsulated within a PA was reported.^126^ The polymer was synthesized based on the click-chemistry between the starting monomers of 4-pentenoic anhydride (4PA), 1,3,5-triallyi-1,3,5-triaxine-2,4,6(1H,3H,5H)-trione (TTT), and 1,4-butane dithiol (BDT). The constructed electronic wireless device is biodegradable, and it is adapted to sense the fluid flow, motion, pH, thermal properties, etc of the physiological activities particularly in the abdomen and extremities, as well as deep within the parenchyma of the brain.

Transient electronic devices are promising for emerging biomedical applications as they are completely bioerodible, and this process occurs in a controlled manner upon serving their function. The use of transient electronics prevents sensitive data leakage, the reduction of electronic waste, secondary surgery to remove electronic implants, etc. Examples of a few transient electronics developed electrophysiological sensors are degradable microwave electronics, disposable sensors, diagnostic brain implants, therapeutic implants, and other environment-friendly degradable devices.^127^ The transience of these electronics undergoes through chemical dissolution process, whereas the device is submerged in aqueous solutions or biofluids for the degradation. Gao et al.^128^ reported novel moisture-triggered physically transient electronic sensors, which do not require any resorption solutions like aqueous solutions or biofluids to control the degradation process of the polymer. Here the degradation process of electronic implants can be regulated by trace amounts of atmospheric moisture. These electronic implants disintegrate completely within a set time period. The moisture-triggered hydrolytic cleavage of the polymer anhydride bond initiates the transient degradation process. Corrosive organic acids of the monomer units are generated as byproducts of the biodegradation process of these implants. These acidic substances can digest the inorganic electronic materials and components. Based on the change of the surface eroding kinetics of the PAs, they can be utilized to design bioelectronics with a tunable time scale of days to weeks. The PA used for this study was synthesized based on the thiol–ene click chemistry between the starting monomers of pentaerythritol tetrakis (mercaptoacetate), 4-pentenoic anhydride and acrylate terminated polyethylene glycol. They have reported that the development of such physically triggered transient mode electronic systems can have potential applications in signal transmission, data storage, etc. Recently, the use of biodegradable PAs as passive encapsulation layers for transient electronics to extend their shelf life has been reported.^129^ The primary duty of the encapsulated layers is to serve as a barrier to prevent the interaction of underlying active materials in the transient electronics with biofluids or water. In this way, the operational lifetime of the transient electronics can be extended. The films of PA, i.e., poly(buthanedithiol 1,3,5-triallyl-1,3,5- triazine-2,4,6(1H,3H,5H)-trionepentenoic anhydride) (PBTPA) were synthesized based on the cross-polymerization of multiarmed containing vinyl monomer i.e., TTT, and 4PA with BDT though thio-ene click chemistry using a mold-photopolymerization technique. The PBTPA films are flexible with high mechanical stability; they undergo dissolution in the phosphate buffer solution at various pH/temperatures through a surface erosion mechanism with little swelling. Controlling the monomer composition and thickness, the water barrier characteristics of PA films on light-emitting diodes with a laser-cut strip of Mg were achieved from a few hours to a week. The films are biocompatible, which does not show any cytotoxic effects in the animal models.

### 6.2 Polyanhydride-Based Nanoparticles for Vaccine Delivery

PA-based nanoparticles have long been used as vaccine delivery vehicles due to their intrinsic adjuvant properties.^130−134^ They increase stability for the vaccine associated antigens, modulate the immune response, and allow for the sustained release of the vaccine associated antigens. The use of such biocompatible nanoparticles can eliminate the use of microbially derived adjuvants, as they suffer from high toxicity and other drawbacks.^19^ Due to the small size and large surface area, the PA-particles are versatile candidates to carry the vaccine components across cellular membranes and deliver them to the specific targeted sites. Moreover, they possess good sustained-release associated characteristics that allow for the development of efficacious single-dose vaccines and eliminate the need for booster shots.^135^

The ideal vaccine delivery system should show robust stability and safeguard the encapsulated vaccine associated antigenic components from the enzymatic degradation. Additionally, an ideal vaccine should be biodegradable and allow for erosion-controlled sustained release. The efficacious vaccine adjuvant platform prepared from PAs showed sustained antibody response to various vaccine associated antigens,^136−140^ increased (CD8^+^) cytotoxic T cell response,^141^ enhanced germinal center formation,^142^ and protection against the targeted bacterial/viral infections.^143−145^ These nano systems are biodegradable, safe and exhibit mild inflammation. Moreover, they have demonstrated that controlling the copolymer composition leads to enhanced cellular uptake,^146,147^ resulting in a composition-dependent immunomodulation.^148,149^

Recently, Narasimhan and co-workers developed PA nanoparticles based on the poly(CPTEG-CPH) (20:80), which is obtained by the melt-condensation polymerization between the starting monomers of CPH and CPTEG. The nanoparticles are prepared through a high-throughput flash-nano precipitation method using an automated chemical robot.^134^ The nanoparticles have several advantages in the biomedical field as vaccine and protein carriers. For example, the nanoparticles were used as carriers in the development of a vaccine against the swine influenza A virus (SwIAV). The authors of the study reported that the PA nanoparticle-based vaccine-induced protective immunity against a heterologous IAV challenge in pigs.^150^ The encapsulation of antigen (KAg) of killed viral influenza virus H1N2-OH10 into the poly(CPTEG-CPH) (20:80) nanoparticles that coencapsulating with KAg and toll-like receptors (TLR)-9 adjuvants (CpG-ODN) is an effective strategy for the intranasal immunization in pigs against swine influenza A virus.^151^ In addition, the potential Influenza A virus vaccines are developed based on pentablock copolymer hydrogels (Pluronic F127-cationicpolydiethylaminoethyl methacrylate) and PA nanoparticles (poly(CPTEG-CPH) (20:80)) to deliver the a recombinant equine H3N8 hemagglutinin trimer (rH33). Its structural stability and antigenicity were studied.^152^ The use of similar nanoparticles as a single-dose combination of nanovaccine provides protection against the seasonal influenza A Virus. For example, the combination PA nano vaccines consisting of both hemagglutinin and nucleoprotein were developed to provide protection against influenza virus infection in aged and young mice.^153^ Similarly, the single-dose combination of poly(CPTEG-CPH) (20:80) encapsulating protective antigen in the combination with cyclic di-GMP (CDG) was developed to induce induces rapid and durable humoral immunity and toxin neutralizing antibody responses against *Bacillus anthracis*.^154^ In another study, similar nanoparticles were used to study the encapsulation, stability and release kinetics of the immunotherapeutic MUC4β-nano vaccine, which is employed to treat pancreatic cancer.^155^*In vivo* mice, experiments with MUC4β loaded nanoparticles verified the immunogenicity of the released MUC4 and its ability to activate dendritic cells and induce adaptive immunity.^156^ In addition, the nanoparticles are also formulated with the H5 mosaic (H5M) vaccine antigen, which can provide the sustained release of the encapsulated antigen for the protection against avian influenza virus in poultry.^157^

In an attempt to develop a better vaccine against Johne’s disease in ruminants, Talaat et al. utilized PA nanoparticles to encapsulate the mycobacterial antigens (whole cell lysate and culture filtrate of M. paratuberculosis), which resulted in the subsequent development of PA-Cf and PA-Lysate nanovaccines.^158^ An increase in the levels of antigen-specific T cell response was observed in mice after vaccination with the PA-based nano vaccines as revealed by immunological assay studies.

### 6.3 Polyanhydrides as Drug, Protein, and Peptide Delivery Carriers

PAs of various sorts have been used as therapeutic delivery vehicles. As discussed previously, Gliadel wafers are used to deliver carmustine (bis-chloroethylnitrosourea, BCNU) for the treatment of brain cancer. The wafers are surgically placed in the tumor, which allows for the localized and controlled delivery of BCNU over several weeks and avoid the unwanted side effects associated with BCNU.^10−14,39^ Antibiotic implants that facilitated local delivery were constructed with poly(eruic acid dimer- sebacic acid) (1:1) copolyanhydride for the treatment of osteomyelitis.^159^ The implant was used to deliver gentamicin sulfate. PAs used in some other drug delivery applications include local anesthetics,^160^ anticancer agents,^161,162^ anticoagulants,^69^ gene therapy candidates,^163^ neuroactive drugs^164^ and other large molecules such as proteins.^165,166^ A detailed review of the drug delivery characteristics of PAs^10^ was published by our group and later updated in 2018.^14^

Poly(esters-anhydrides) in the form of microspheres and nanoparticles have been used for drug delivery applications for several years now. For example, Uhrich et al. reported the design and use of salicylate-based poly(anhydride-ester) microspheres.^167^ The microspheres are prepared based on the oil-in-water single emulsion solvent evaporation method using three salicylate-based PAEs (heteroatomic, linear aliphatic or branched aliphatic moiety). These microspheres allowed the controlled release of salicylic acid *in vitro* with tunable release characteristics in the range of days to months. Furthermore, the same microspheres were also employed as carriers to deliver insulin under *in vitro* conditions.^168^ Several studies describe the synthesis and use of insulin loaded microsphere formulations which were prepared using the water/oil/water double-emulsion solvent evaporation technique. The synthesized microspheres were uniform in size, possessed a smooth surface, and had high protein encapsulation efficiencies. Insulin was released *in vitro* for 15 days without any sign of aggregation or unfolding of the secondary structure. In continuation, Jaszcz et al. reported the development of microspheres of poly(ester-anhydrides) with pendant allyl groups using the solvent evaporation technique. The poly(ester-anhydrides) were obtained by polycondensation of oligo(3-allyloxy-1,2-propylene succinate) terminated with carboxyl groups (OSAGE) with SA or dodecane dicarboxylic acid (DDC). Cross-linking photopolymerization of microparticles with pendant allyl group lead to the formation of porous structures. Porous microparticles were used to deliver substances like rhodamine B, p-nitroaniline, and piroxicam.^169−171^

Recently, the Narasimhan group also contributed significantly to the use of PA-microspheres or nanoparticles for the sustained release of proteins. As discussed previously, they have synthesized PA-based microspheres and nanoparticles based on the combinations of SA, CPH, and CPTEG monomers. In one of the studies, the protein “ovalbumin” was encapsulated in the PA-microspheres based on two nonaqueous methods as solid/oil/oil double emulsion technique, and cryogenic atomization.^172^ The studies reveal that the formulated microspheres showed enhanced stability for the ovalbumin structure. In another study, they also used similar PA microspheres for the loading and release of Pneumococcal surface protein A (PspA).^166^ Similarly, the PA-based nanoparticles were used to encapsulate ovalbumin, and the release kinetics with respect to different concentrations of surfactant (Span 80) was analyzed. The studies conclude that the surfactant does not exert any significant effect on the release kinetics of ovalbumin, however, the authors noted that the surfactant concentration altered protein encapsulation efficacy.^134^ In all these studies the activity of proteins can be maintained effectively even after sustained release from PA-nanoparticles/microspheres. These features make the PA-particles an efficient candidate for the delivery of proteins.

The bioactive PA microspheres prepared from drug molecules were also reported for therapeutic delivery. Recently, Niewolik et al. reported the synthesis of bioactive PA copolymers involving melt polymerization of the acid-functionalized butulin and PEG in the presence of acetic anhydride.^80^ Butulin is a bioactive natural compound that exhibits broad spectrum antibacterial activity.^173^ These microspheres released the drug in a controlled manner in the PBS at 37 °C due to the hydrolytic cleavage of the polymer anhydride bond. The bioavailability of the microspheres could be adjusted with changing the PEG composition in the copolymer structure.

The development of biodegradable amphiphilic polymeric micellar nanoparticles is an attractive approach that is used to deliver hydrophobic drugs. Such nanoparticles are usually prepared from copolymers consisting of both hydrophobic and hydrophilic blocks. For example, Zhai et al. reported novel amphiphilic block copolymer nanoparticles of mPEG-bP(RA-SA)-b-mPEG that are composed of the hydrophilic poly(PEG) shell and SA-RA blocks as the hydrophobic core.^174^ They used a nano micellar system to deliver the hydrophobic anticancer drug paclitaxel. The drug was encapsulated in the hydrophobic core matrix of the nanoparticles. The loading of the drug into the micellar nanoparticles is dependent on the RA-SA composition in the polymer matrix. The *in vitro* release of the drug from the nanoparticles exhibited a biphasic profile with zero-order release kinetics. Another study by Zhou et al. demonstrated the use of poly(PEG:CPP:SA) terpolymers as a promising thermosensitive amphiphilic nanocarrier micelle for the potential delivery of the anticancer drug “doxorubicin hydrochloride”.^175^ The terpolymer was constructed based on the combination of hydrophilic poly(PEG) and hydrophobic poly(CPP-SA) blocks. The developed nanomicelles were thermoresponsive, and they underwent a phase transition from solution to gel as the temperature increased from the RT to body temperature. Based on the cytotoxicity studies they concluded that the terpolymer micelles were biocompatible and were able to maintain the release of the drug in a sustained manner. The same research group reported another design of an amphiphilic PEG–PA micelle ((mPEG)-SS-(CPP-SA)), which showed redox or acid-mediated delivery of curcumin.^176^ The prepared polymer nano micelles were sensitive to reducing agents (glutathione) in acidic environments like those observed at tumor sites, which resulted in the cleavage of the micelle’s structure and rapid release of the drug. These nanomicelles are safe, biocompatible, and exhibited better therapeutic effects *in vivo.*

Finally, various kinds of PAs were developed in the form of polyactives for the controlled release of bioactive small organic compounds and drugs. The polyactives have several advantages compared to the other traditional and physical admixing-based formulations. They are usually synthesized by the chemical incorporation of small bioactive molecules/drugs into the biodegradable polymer matrix. This approach would confer properties such as high drug loading, enhanced processability and better controlled release kinetics to the polymer.^118^ Poly(anhydride-ester) based polyactives can also be employed as prodrug platforms for the controlled release of various therapeutics and bioactive small organic molecules including antioxidants,^177−181^ anti-inflammatory drugs,^182,183^ antiseptics,^181,184^ antibiotics,^185^ and antimicrobial drugs.^186^ Recently, Madras et al. also reported several poly(anhydride-esters) that contained bioactive molecules such as salicylic acid and aspirin in the polymer main chain.^187−190^ They studied the release kinetics associated with the hydrolytic degradation of the polymer matrix and subsequently investigated their anti-inflammatory and antibacterial activities.

## 7 Challenges and Alternative Routes of Polyanhydride Biomaterials

In the last section, we have highlighted the advantageous of PAs used in the biomedical field as drugs, vaccines and protein delivery carriers as well as in the bioelectronics. The class of PAs possesses a few significant advantages such as surface erosion, fast hydrolytic degradation, and strong mechanical properties compared to other classes of synthetic polymers (ex: polyesters).^10−14^ Such properties keep PAs as first-choice biomaterials for drug/therapeutic delivery. However, due to their poor stability toward moisture, their applicability in the biomedical field is hampered.^10−14^ Currently, most of the available PAs are not stable at room temperatures for a long time due to interaction with room atmospheric moisture. This can lead to decomposition of the anhydride bond, thus changes in the polymer molecular weight arise.^11,34,45,46^ The unstable biopolymers affect drug release kinetics. Hence, they can be stored under inert atmospheres at low temperatures.^108^ Other factors influencing the biomedical properties of the PAs include their physicochemical (example: melting temperature, viscosity, solubility, crystallinity, hydrophilicity) and mechanical properties (example: tensile strength, etc.). The PAs with poor crystallinity, high hydrophilicity,^120−122^ and poor mechanical properties^65,85,86^ affect their surface erosion properties due to the fast degradation of the anhydride bond. The high melting PAs lead to difficulty in fabrication into films or microspheres/nanoparticles.^57^ Therefore, the PAs with suitable physicochemical and mechanical properties are a considerable aspect. Moreover, the physical status of the PAs also affects their biomedical properties. For example, PAs in the form of microspheres and nanoparticles are potential for the intravenous delivery of various drugs, vaccines, proteins, and other therapeutics.^153,154,158,161,166^ While the PAs in the form of biomedical devices and gels are efficient for local delivery of drugs and other therapeutics.^12,14,48^ However, the loading of drugs/vaccines into the PAs microspheres and nanoparticles in the required dimensions is a challenging approach.^103,134,169^ Factors influencing the drug loading, its stability and its controlled release from the microspheres/nanoparticles are needs to be taken care of while developing PA-based intravenous drug delivery platforms. Another problem includes the use of organic solvents during the preparation of microspheres/nanoparticles. This leads to chance of trapped organic solvents in the core of the microspheres/nanoparticles, which might cause harmful effects on the biological system.

To address the key challenges associated with PAs, the proper selection of suitable monomers and changing their composition ratios during polymer synthesis is required. This approach would lead to the design of novel PAs with suitable physicochemical and mechanical properties. The PAs with high crystallinity, high hydrophobicity or low hydrophilicity, and high tensile strength allows prolonged and controlled drug release kinetics. Such properties can be altered by playing with hydrophobic content in the polymer structure. The inclusion of hydrophobic units alternatively and placing them adjacent to the anhydride bond controls the degradation rate.^43,49,54^ The hydrophobic chain shields the anhydride bond, thus controlling its cleavage from water in such a way stable PAs could be developed.^43,44,54,55^ The problem associated with poor drug encapsulation efficacy, stability and drug release kinetics of microspheres/nanoparticles is also addressed by increasing their hydrophobicity.^56^ One significant approach is the use of biocompatible fatty acids as hydrophobic monomer segments during the PA synthesis; this can lead to room temperature stable PAs with low melting nature.^43,44,54,55^ In addition, the degraded metabolites of fatty acid-based PAs are eco-friendly to the body and can be easily eliminated. The development of novel synthetic methodologies based on the use of eco-friendly metal-based catalysts (example: CaO, Ca(OH)_2_, and CaCO_3_), optimizing the concentration of acetic anhydride ratios, reaction time, temperature, etc. is another way to produce a stable and high molecular weight PAs.^55,49^

## 8 Conclusion and Outlook

Polyanhydrides (PAs) have long been developed and applied as delivery vehicles for low to high-molecular-weight drugs, vaccines, proteins, peptides, and nucleotides. For this purpose, PA carriers in the form of micro/nanoparticles, microspheres, injectable pasty formulations, etc., have been developed. PAs have found applications in the medical implant sector as well. The primary method for PA synthesis is melt condensation of diacid monomers in the presence of acetic anhydride. Several factors influencing the polymer degradation rate include pH of the medium, monomer chemical structures and its orientation in the polymer matrix, crystallinity, and type of drug being loaded. The degradation rate of PAs can be manipulated by changing the hydrophilic and hydrophobic monomer compositions in the copolymer. Prolonged drug release was achieved with hydrophobic PAs. Fatty-acid monomers were used for the development of room temperature stable PAs with enhanced shelf-lives. They give rise to pasty polymers that can be used to develop easily injectable formulations with a variety of drugs and allows for their sustained release. Various synthetic methodologies were reported for producing different classes of aromatic, aliphatic, aliphatic-aromatic, cross-linked, and fatty-acid-based PAs, which possess a wide variety of molecular structures and exhibit different physicochemical and mechanical properties. Among them, a few PA-drug delivery carriers solved major problems in drug/vaccine delivery as described below:

- Polyanydrides address the problems related to the delivery of toxic drugs. Gliadel wafer constructed with poly(CPP-SA) was developed for the local delivery of BCNU for treating brain cancer. Gliadel wafers are placed in the tumor bed after surgery, which locally releases BCNU over several weeks. This approach avoids BCNU systemic toxicity as minimal BCNU is distributed to other organs.^13^
- The clinically tested implant “septacin” was developed for the sustained local delivery of the antibiotic drug, gentamicin sulfate, for the treatment of osteomyelitis. In this application, the PA copolymer of erucic acid dimer and sebacic acid was used for the construction of molded Septacin linked beads.^159^
- Due to the sustained and controlled release characteristics of PA-based delivery carriers facilitates the development of single-dose combination vaccines, thereby eliminating multiple shots. For example, the microspheres and nanoparticles of poly(CPTEG-CPH) (20:80) were developed as single-dose combination vaccine delivery carriers for protection against seasonal influenza A Virus.^139,143,153^ They increase stability for the vaccine-associated antigens, modulate the immune response, and allow for the sustained release of the vaccine-associated antigens.
- The anhydride formation chemistry allows the development of diacid-therapeutics into PA-prodrugs; therefore, the chance of the inflammatory response from degraded products of the PA-prodrugs can be minimized with improved biocompatibility.^77−81^

However, only Gliadel wafer is currently in clinical use. Efforts are ongoing to clinically develop the ricinoleic acid based injectable PAs for the delivery of anticancer agents, antibiotics, and biological drugs. PA-based drug delivery systems will result a proliferation of clinical products arising from this sector in the years to come.

## References

[ref1] Doppalapudi S.; Jain A.; Khan W.; Domb A. J. Biodegradable Polymers-an Overview. Polym. Adv. Technol. 2014, 25, 427–435. 10.1002/pat.3305.

[ref2] Kumar N.; Ravikumar M. N. V.; Domb A. J. Biodegradable Block Copolymers. Adv. Drug Delivery Rev. 2001, 53, 23–44. 10.1016/S0169-409X(01)00219-8. 11733116

[ref3] Basu A.; Kunduru K. R.; Abtew E.; Domb A. J. Polysaccharide-Based Conjugates for Biomedical Applications. Bioconjugate Chem. 2015, 26, 1396–1412. 10.1021/acs.bioconjchem.5b00242. 26106905

[ref4] Guruprasad Reddy P.; Domb A. J. Formation of Micro/nanoparticles and Microspheres from Polyesters by Dispersion Ring-opening Polymerization. Polym. Adv. Technol. 2021, 32, 3835–3856. 10.1002/pat.5476.

[ref5] Arun Y.; Ghosh R.; Domb A. J. Biodegradable Hydrophobic Injectable Polymers for Drug Delivery and Regenerative Medicine. Adv. Funct. Mater. 2021, 31, 2010284 10.1002/adfm.202010284.

[ref6] Ulery B. D.; Nair L. S.; Laurencin C. T. Biomedical Applications of Biodegradable Polymers. J. Polym. Sci., Part B: Polym. Phys. 2011, 49, 832–864. 10.1002/polb.22259. PMC3136871 21769165

[ref7] Shah T. V.; Vasava D. V. A Glimpse of Biodegradable Polymers and Their Biomedical Applications. e-Polym. 2019, 19, 385–410. 10.1515/epoly-2019-0041.

[ref8] Sung Y. K.; Kim S. W. Recent Advances in Polymeric Drug Delivery Systems. Biomater. Res. 2020, 24, 1–12. 10.1186/s40824-020-00190-7. 32537239 PMC7285724

[ref9] Kumar R.; Butreddy A.; Kommineni N.; Reddy P. G.; Bunekar N.; Sarkar C.; Dutt S.; Mishra V. K.; Aadil K. R.; Mishra Y. K.; Oupicky D.; Kaushik A. Lignin: Drug/Gene Delivery and Tissue Engineering Applications. Int. J. Nanomed. 2021, 16, 2419–2441. 10.2147/IJN.S303462. PMC8009556 33814908

[ref10] Kumar N.; Langer R. S.; Domb A. J. Polyanhydrides: An Overview. Adv. Drug Delivery Rev. 2002, 54, 889–910. 10.1016/S0169-409X(02)00050-9. 12384314

[ref11] Göpferich A.; Tessmar J. Polyanhydride Degradation and Erosion. Adv. Drug Delivery Rev. 2002, 54, 911–931. 10.1016/S0169-409X(02)00051-0. 12384315

[ref12] Tamada J.; Langer R. The Development of Polyanhydrides for Drug Delivery Applications. J. Biomater. Sci., Polym. Ed. 1992, 3, 315–353. 10.1163/156856292X00402. 1350734

[ref13] Poetz K. L.; Shipp D. A. Polyanhydrides: Synthesis, Properties, and Applications. Aust. J. Chem. 2016, 69, 1223–1239. 10.1071/CH16144.

[ref14] Basu A.; Domb A. J. Recent Advances in Polyanhydride Based Biomaterials. Adv. Mater. 2018, 30, 1706815 10.1002/adma.201706815. 29707879

[ref15] Lakshmi S.; Katti D. S.; Laurencin C. T. Biodegradable Polyphosphazenes for Drug Delivery Applications. Adv. Drug Delivery Rev. 2003, 55, 467–482. 10.1016/S0169-409X(03)00039-5. 12706046

[ref16] Vauthier C.; Dubernet C.; Fattal E.; Alphandary H. P.; Couvreur P. Poly(alkylcyanoacrylates) as Biodegradable Materials for Bio-medical Applications. Adv. Drug Delivery Rev. 2003, 55, 519–548. 10.1016/S0169-409X(03)00041-3. 12706049

[ref17] Numata K. Poly(amino acid)s/polypeptides as Potential Functional and Structural Materials. Polym. J. 2015, 47, 537–545. 10.1038/pj.2015.35.

[ref18] Kutikov A. B.; Song J. Biodegradable PEG-Based Amphiphilic Block Copolymers for Tissue Engineering Applications. ACS Biomater. Sci. Eng. 2015, 1, 463–480. 10.1021/acsbiomaterials.5b00122. 27175443 PMC4860614

[ref19] Ghadi R.; Muntimadugu E.; Domb A. J.; Khan W.; Zhang X. 5 - Synthetic biodegradable medical polymer: Polyanhydrides. Science and Principles of Biodegradable and Bioresorbable Medical Polymers Materials and Properties, Zhang X., Ed.; Elsevier: Wood head Publishing, 2017; pp 153–188. 10.1016/B978-0-08-100372-5.00005-2.

[ref20] Murthy N.; Wilson S.; Sy J. C. 9.28-Biodegradation of Polymers. Polymer Science: A Comprehensive Reference; Matyjaszewski K., Möller M., Eds.; Elsevier, 2012; Vol. 9, pp 547–560. 10.1016/B978-0-444-53349-4.00240-5.

[ref21] Domb A. J.; Amselem S.; Shah J.; Maniar M. Polyanhydrides: Synthesis and characterization. Advances in Polymer Science; Langer R. S., Peppas N. A., Eds.; Springer: Berlin, Heidelberg, 1993; Vol. 107, pp 94–141. 10.1007/BFb0027552.

[ref22] Ghadi R.; Muntimadugu E.; Khan W.; Domb A. J. Polyanhyydrides, Polymers for Biomedicine: Synthesis, Characterization, and Applications; Scholz C., Ed.; John Wiley & Sons, Inc, Hoboken, NJ, 2017; pp 121–148. 10.1002/9781118967904.ch5.

[ref23] Eameema M.; Duvvuri L.S. S.; Khan W.; Domb A. J. Chapter 10 – Polyanhydrides. Natural and synthetic biomedical polymers; Kumbar S. G., Laurencin C. T., Deng M., Eds.; Elsevier: San Diego, USA, 2014; pp 181–192. 10.1016/B978-0-12-396983-5.00010-7.

[ref24] Bucher J. E.; Slade W. C. The Anhydrides of Isophthalic and Terephthalic Acids. J. Am. Chem. Soc. 1909, 31, 1319–1321. 10.1021/ja01942a009.

[ref25] Hill J. W. Studies on Polymerization and Ring Formation.VI. Adipic Anhydride. J. Am. Chem. Soc. 1930, 52, 4110–4114. 10.1021/ja01373a053.

[ref26] Hill J. W.; Carothers W. H. Studies of Polymerization and Ring Formation. XIV. A Linear Super Polyanhydride and a Cyclic Dimeric Anhydride from Sebacic acid. J. Am. Chem. Soc. 1932, 54, 1569–1579. 10.1021/ja01343a050.

[ref27] Carothers W. H.; Hill J. W. Studies of Polymerization and Ring Formation. XV. Artificial Fibers from Synthetic Linear Condensation Super polymers. J. Am. Chem. Soc. 1932, 54, 1579–1587. 10.1021/ja01343a051.

[ref28] Conix A. Aromatic Polyanhydrides, A New class of High Melting Fiber-forming Polymers. J. Polym. Sci. 1958, 29, 343–353. 10.1002/pol.1958.1202912002.

[ref29] Yoda N. Synthesis of Polyanhydrides. II. New Aromatic Polyanhydrides with High Melting Points and Fiber-forming Properties. Macromol. Chem. Phys. 1959, 32, 1–12. 10.1002/macp.1959.020320101.

[ref30] Yoda N. Synthesis of Polyanhydrides. XI. Synthesis and Properties of New Polythioether Polyanhydrides. Macromol. Chem. Phys. 1962, 56, 36–54. 10.1002/macp.1962.020560103.

[ref31] Yoda N. Synthesis of Polyanhydrides. III. Polyanhydrides of Five-membered Heterocyclic Dibasic Acids. Macromol. Chem. Phys. 1962, 55, 174–190. 10.1002/macp.1962.020550114.

[ref32] Yoda N. Synthesis of Polyanhydrides. X. Mixed Anhydrides of Aromatic and Five-membered Heterocyclic Dibasic Acids. Macromol. Chem. Phys. 1962, 56, 10–35. 10.1002/macp.1962.020560102.

[ref33] Rosen H. B.; Chang J.; Wnek G. E.; Linhardt R. J.; Langer R. Bio-erodible Polyanhydrides for Controlled Drug Delivery. Biomaterials. 1983, 4, 131–133. 10.1016/0142-9612(83)90054-6. 6860755

[ref34] Domb A. J.; Langer R. S. Polyanhydrides with Improved Hydrolytic Degradation Properties. US PatentUS4,857,311, August 15, 1989.

[ref35] Langer R. S.; Domb A. J.; Laurencin C. T. Controlled Drug Delivery High Molecular Weight Polyanhydrides. US patent US4,888,176, December 19, 1989.

[ref36] Domb A. J.; Langer R. S. High Molecular Weight Polyanhydride and Preparation Thereof. US PatentUS4,757,128, July 12, 1988.

[ref37] Domb A. J.; Langer R. S. Unsaturated Polyanhydrides. US PatentUS5,019,379, May 28, 1991.

[ref38] Domb A. J.; Maniar M. Branched Polyanhydrides, US PatentUS5,175,235, December 29, 1992.

[ref39] Brem H.; Ewend M. G.; Piantadosi S.; Greenhoot J.; Burger P. C.; Sisti M. The Safety of Interstitial Chemotherapy with BCNU-loaded Polymer Followed by Radiation Therapy in the Treatment of Newly Diagnosed Malignant Gliomas: Phase I Trial. J. Neuro-Oncol. 1995, 26, 111–123. 10.1007/BF01060217. 8787853

[ref40] Brem H.; Lawson H. C. The Development of New Brain Tumor Therapy Utilizing the Local and Sustained Delivery of Chemotherapeutic Agents from Biodegradable Polymers. Cancer. 1999, 86, 197–199. 10.1002/(SICI)1097-0142(19990715)86:2<197::AID-CNCR2>3.0.CO;2-6. 10421254

[ref41] Olivi A.; Bruce J.; Saris S.; Engelhard H.; Judy K.; Kelly D. The NABTT CNS Consortium and Guilford Pharmaceuticals Inc. Phase I study of escalating doses of interstitial BCNU administered via wafer in patients with recurrent malignant glioma (MG). Proc. Am. Soc. Clin. Oncol. 1998, 17, 387.

[ref42] Krasko M. Y.; Shikanov A.; Ezra A.; Domb A. J. Poly(ester anhydride)s Prepared by the Insertion of Ricinoleic acid into Poly(sebacic acid). J. Polym. Sci., Part A: Polym. Chem. 2003, 41, 1059–1069. 10.1002/pola.10651.

[ref43] Haim-Zada M.; Basu A.; Hagigit T.; Schlinger R.; Grishko M.; Kraminsky A.; Hanuka E.; Domb A. J. Alternating Poly(ester-anhydride) by Insertion Polycondensation. Biomacromolecules. 2016, 17, 2253–2259. 10.1021/acs.biomac.6b00523. 27198864

[ref44] Haim-Zada M.; Basu A.; Hagigit T.; Schlinger R.; Grishko M.; Kraminsky A.; Hanuka E.; Domb A. J. Stable Polyanhydride Synthesized from Sebacic Acid and Ricinoleic Acid. J. Controlled Release 2017, 257, 156–162. 10.1016/j.jconrel.2016.04.036. 27126904

[ref45] Krasko M. Y.; Domb A. J. Hydrolytic Degradation of Ricinoleic-Sebacic-Ester-Anhydride Copolymers. Biomacromolecules. 2005, 6, 1877–1884. 10.1021/bm049228v. 16004424

[ref46] Vaisman B.; Ickowicz D. E.; Abtew E.; Zada M. H.; Shikanov A.; Domb A. J. In Vivo Degradation and Elimination of Injectable Ricinoleic Acid-Based Poly(ester-anhydride). Biomacromolecules. 2013, 14, 1465–1473. 10.1021/bm4001475. 23530926

[ref47] Popilski H.; Abtew E.; Schwendeman S.; Domb A.; Stepensky D. Efficacy of Paclitaxel/dexamethasone Intra-tumoral Delivery in Treating Orthotopic Mouse Breast Cancer. J. Controlled Release 2018, 279, 1–7. 10.1016/j.jconrel.2018.04.010. 29654797

[ref48] Krasko M. Y.; Golenser J.; Nyska M.; Brin Y. S.; Domb A. J.; et al. Gentamicin Extended Release from an Injectable Polymeric Implant. J. Controlled Release 2007, 117, 90–96. 10.1016/j.jconrel.2006.10.010. 17150275

[ref49] Ghosh R.; Siman P.; Domb A. J. Poly(ester-anhydrides) with Controlled Molecular Weight and Structure. Polym. Adv. Technol. 2022, 33, 3774–3781. 10.1002/pat.5711.

[ref50] Korhonen H.; Helminen A. O.; Seppälä J. V. Synthesis of Poly(ester-anhydrides) Based on Different Polyester Precursors. Macromol. Chem. Phys. 2004, 205, 937–945. 10.1002/macp.200300208.

[ref51] Storey R. F.; Taylor A. E. Synthesis of Novel Biodegradable Poly(Ester-Anhydride)s. J. Macromol. Sci., Part A: Pure Appl.Chem. 1997, 34 (2), 265–280. 10.1080/10601329708014954.

[ref52] Pfeifer B. A.; Burdick J. A.; Langer R. Formulation and Surface Modification of Poly(ester-anhydride) Micro- and Nanospheres. Biomaterials. 2005, 26, 117–124. 10.1016/j.biomaterials.2004.02.015. 15207458

[ref53] Pfeifer B. A.; Burdick J. A.; Little S. R.; Langer R. Poly(ester-anhydride): Poly(β-amino ester) Micro- and Nanospheres: DNA Encapsulation and Cellular Transfection. Int. J. Pharm. 2005, 304, 210–219. 10.1016/j.ijpharm.2005.08.001. 16174553

[ref54] Arun Y.; Ghosh R.; Domb A. J. Poly(ester-anhydrides) Derived from Esters of Hydroxy Acid and Cyclic Anhydrides. Biomacromolecules. 2022, 23, 3417–3428. 10.1021/acs.biomac.2c00542. 35881559 PMC9516692

[ref55] Ghosh R.; Arun Y.; Siman P.; Domb A. J. Synthesis of Aliphatic Polyanhydrides with Controllable and Reproducible Molecular Weight. Pharmaceutics. 2022, 14, 1403 10.3390/pharmaceutics14071403. 35890298 PMC9325212

[ref56] Zada M. H.; Kubek M.; Khan W.; Kumar A.; Domb A. Dispersible Hydrolytically Sensitive Nanoparticles for Nasal Delivery of Thyrotropin Releasing Hormone (TRH). J. Controlled Release 2019, 295, 278–289. 10.1016/j.jconrel.2018.12.050. 30610951

[ref57] Domb A. J. Synthesis and Characterization of Biodegradable Aromatic Anhydride Copolymers. Macromolecules 1992, 25, 12–17. 10.1021/ma00027a003.

[ref58] Leong K. W.; Brott B. C.; Langer R. Bioerodible Polyanhydrides as Drug-carrier Matrices. I: Characterization, Degradation, and Release Characteristics. J. Biomed. Mater. Res. 1985, 19, 941–955. 10.1002/jbm.820190806. 3880353

[ref59] Snyder S. S.; Anastasiou T. J.; Uhrich K. E. In vitro Degradation of an Aromatic Polyanhydride with Enhanced Thermal Properties. Polym. Degrad. Stab. 2015, 115, 70–76. 10.1016/j.polymdegradstab.2015.02.002. 25870460 PMC4392399

[ref60] Torres M. P.; Vogel B. M.; Narasimhan B.; Mallapragada S. K. Synthesis and Characterization of Novel Polyanhydrides with Tailored Erosion Mechanisms. J. Biomed. Mater. Res., Part A 2006, 76 (1), 102–10. 10.1002/jbm.a.30510. 16138330

[ref61] Campo C.; Anastasiou T.; Uhrich K. Polyanhydrides: The Effects of Ring Substitution Changes on Polymer Properties. Polym. Bull. 1999, 42, 61–68. 10.1007/s002890050435.

[ref62] Ron E.; Turek T.; Mathiowitz E.; Chasin M.; Hageman M.; Langer R. Controlled Release of Polypeptides from Polyanhydrides. Proc. Natl. Acad. Sci. U. S. A. 1993, 90, 4176–4180. 10.1073/pnas.90.9.4176. 8483931 PMC46469

[ref63] Leong K. W.; Simonte V.; Langer R. Synthesis of Polyanhydrides: Melt-Polycondensation, Dehydrochlorination, and Dehydrative Coupling. Macromolecules. 1987, 20, 705–712. 10.1021/ma00170a001.

[ref64] Domb A. J.; Nudelman R. *In Vivo* and *in Vitro* Elimination of Aliphatic Polyanhydrides. Biomaterials 1995, 16, 319–323. 10.1016/0142-9612(95)93260-K. 7772672

[ref65] Domb A. J.; Langer R. Polyanhydrides. I. Preparation of High Molecular Weight Polyanhydrides. J. Polym. Sci., Part A: Polym. Chem. 1987, 25, 3373–3386. 10.1002/pola.1987.080251217.

[ref66] Albertsson A. C.; Carlfors J.; Sturesson C. Preparation and Characterisation of Poly(adipic anhydride) Microspheres for Ocular Drug Delivery. J. Appl. Polym. Sci. 1996, 62, 695–705. 10.1002/(SICI)1097-4628(19961024)62:4<695::AID-APP13>3.0.CO;2-V.

[ref67] Shelke N. B.; Aminabhavi T. M. Synthesis and Characterization of Novel Poly(sebacic anhydride-co-Pluronic F68/F127) Biopolymeric Microspheres for the Controlled Release of Nifedipine. Int. J. Pharm. 2007, 345, 51–58. 10.1016/j.ijpharm.2007.05.036. 17616283

[ref68] Domb A. J.; Mathiowitz E.; Ron E.; Giannos S.; Langer R. Polyanhydrides. IV. Unsaturated and Crosslinked Polyanhydrides. J. Polym. Sci., Part A: Polym. Chem. 1991, 29, 571–579. 10.1002/pola.1991.080290413.

[ref69] Chickering D.; Jacob J.; Mathiowitz E. Poly(fumaric-co-sebacic) Microspheres as Oral Drug Delivery Systems. Biotechnol. Bioeng. 1996, 52, 96–101. 10.1002/(SICI)1097-0290(19961005)52:1<96::AID-BIT9>3.0.CO;2-U. 18629855

[ref70] Sandor M.; Bailey N. A.; Mathiowitz E. Characterization of Polyanhydride Microsphere Degradation by DSC. Polymer. 2002, 43, 279–288. 10.1016/S0032-3861(01)00612-7.

[ref71] Huarte J.; Espuelas S.; Lai Y.; He B.; Tang J.; Irache J. M. Oral Delivery of Camptothecin Using Cyclodextrin/poly(anhydride) Nanoparticles. Int. J. Pharm. 2016, 506, 116–128. 10.1016/j.ijpharm.2016.04.045. 27102993

[ref72] Agüeros M.; Ruiz-Gatón L.; Vauthier C.; Bouchemal K.; Espuelas S.; Ponchel G.; Irache J. M. Combined Hydroxypropyl-β-cyclodextrin and Poly(anhydride) Nanoparticles Improve the Oral Permeability of Paclitaxel. Eur. J. Pharm. Sci. 2009, 38 (4), 405–413. 10.1016/j.ejps.2009.09.010. 19765652

[ref73] Calvo J.; Lavandera M.; Agüeros J. M.; et al. Irache, Cyclodextrin/poly(anhydride) Nanoparticles as Drug Carriers for the Oral Delivery of Atovaquone. Biomed. Microdevices. 2011, 13 (6), 1015–1025. 10.1007/s10544-011-9571-1. 21773725

[ref74] Lucio D.; Martinez-Oharriz M. C.; Gu Z.; He Y.; Aranaz P.; Vizmanos J. L.; Irache J. M. Cyclodextrin-Grafted Poly(anhydride) Nanoparticles for Oral Glibenclamide Administration. In Vivo Evaluation Using C. Elegans. Int. J. Pharm. 2018, 547, 97–105. 10.1016/j.ijpharm.2018.05.064. 29842888

[ref75] Lucio D.; Martínez-Ohárriza M. C.; González-Navarro C. J.; Herrera D. N.; González-Gaitano G.; Radulescu A.; Irached J. M. Coencapsulation of Cyclodextrins into Poly(anhydride) Nanoparticles to Improve the Oral Administration of Glibenclamide. A Screening on C. Elegans. Colloids Surf. B: Biointerfaces. 2018, 163, 64–72. 10.1016/j.colsurfb.2017.12.038. 29277019

[ref76] Al-Heibshy F. N. S.; Basaran E.; Arslan R.; Ozturk N.; Vural I.; Demirel M. Preparation, Characterization and Pharmacokinetic Evaluation of Rosuvastatin Calcium Incorporated Cyclodextrin-Polyanhydride Nanoparticles. Drug Dev. Ind. Pharm. 2019, 45, 1635–1645. 10.1080/03639045.2019.1648501. 31342792

[ref77] Niewolik D.; Krukiewicz K.; Bednarczyk-Cwynar B.; Ruszkowskic P.; Jaszcz K. Novel Polymeric Derivatives of Betulin with Anticancer Activity. RSC Adv. 2019, 9, 20892–20900. 10.1039/C9RA03326B. 35515533 PMC9065995

[ref78] Niewolik D.; Dzido G.; Jaszcz K. Studies on the Preparation of Nanoparticles from Betulin-Based Polyanhydrides. Eng. Proc. 2021, 11, 10.

[ref79] Niewolik D.; Bednarczyk-Cwynar B.; Ruszkowski P.; Jaszcz K. Novel Biodegradable Polyanhydrides Based on BetulinDisuccinate and Sebacic Acid for Medical Purpose. Proceedings. 2020, 67, 17.

[ref80] Niewolik D.; Bednarczyk-Cwynar B.; Ruszkowski P.; Sosnowski T. R.; Jaszcz K. Bioactive Betulin and PEG Based Polyanhydrides for Use in Drug Delivery Systems. Int. J. Mol. Sci. 2021, 22, 1090 10.3390/ijms22031090. 33499242 PMC7865682

[ref81] Niewolik D.; Bednarczyk-Cwynar B.; Ruszkowski P.; Kazek-Kesik A.; Dzido G.; Jaszcz K. Biodegradable and Bioactive Carriers Based on Poly(betulindisuccinate-co-sebacic Acid) for Rifampicin Delivery. Pharmaceutics. 2022, 14, 579 10.3390/pharmaceutics14030579. 35335954 PMC8953921

[ref82] Ruiz-Gaton L.; Espuelas S.; Larraneta E.; Reviakine I.; Yate L. A.; Irache J. M. Pegylated Poly(anhydride) Nanoparticles for Oral Delivery of Docetaxel. Eur. J. Pharm. Sci. 2018, 118, 165–175. 10.1016/j.ejps.2018.03.028. 29597043

[ref83] Ruiz-Gatón L.; Espuelas S.; Huarte J.; Larrañeta E.; Martin-Arbella N.; Irache J. M. Nanoparticles from Gantrez® AN-poly(ethylene glycol) Conjugates as Carriers for Oral Delivery of Docetaxel. Int. J. Pharm. 2019, 571, 118699 10.1016/j.ijpharm.2019.118699. 31536764

[ref84] Santos C. A.; Freedman B. D.; Leach K. J.; Press D. L.; Scarpulla M.; Mathiowitz E. Poly(fumaric–co-sebacic anhydride) A Degradation Study as Evaluated by FTIR, DSC, GPC and X-ray Diffraction. J. Controlled Release 1999, 60, 11–22. 10.1016/S0168-3659(99)00016-4. 10370167

[ref85] Anseth K. S.; Quick D. J. Polymerizations of Multifunctional Anhydride Monomers to Form Highly Crosslinked Degradable Networks. Macromol. Rapid Commun. 2001, 22, 564–572. 10.1002/1521-3927(20010501)22:8<564::AID-MARC564>3.0.CO;2-S.

[ref86] Burkoth A. K.; Anseth K. S. A Review of Photo Crosslinked Polyanhydrides: In-situ Forming Degradable networks. Biomaterials. 2000, 21, 2395–2404. 10.1016/S0142-9612(00)00107-1. 11055287

[ref87] Shipp D. A.; McQuinn C. W.; Rutherglen B. G.; Mc Bath R. A. Elastomeric and Degradable Polyanhydride Network Polymers by Step-growth Thiol-ene Photopolymerization. ChemComm. 2009, 6415–6417. 10.1039/b911557a. 19841794

[ref88] Rutherglen B. G.; McBath R. A.; Huang Y. L.; Shipp D. A. Polyanhydride Networks from Thiol-Ene Polymerizations. Macromolecules. 2010, 43, 10297–10303. 10.1021/ma102287v.

[ref89] Poetz K. L.; Mohammed H. S.; Snyder B. L.; Liddil G.; Samways D. S. K.; Shipp D. A. Photopolymerized Cross-Linked Thiol–Ene Polyanhydrides: Erosion, Release, and Toxicity Studies. Biomacromolecule. 2014, 15, 2573–2582. 10.1021/bm500420q. 24848134

[ref90] Poetz K. L.; Mohammed H. S.; Shipp D. A. Surface Eroding, Semi-crystalline Polyanhydrides via Thiol–Ene “Click” Photopolymerization. Biomacromolecules. 2015, 16, 1650–1659. 10.1021/acs.biomac.5b00280. 25867183

[ref91] Mohammed H. S.; Snyder B. L.; Samways D. S. K.; Shipp D. A. Quantitative and Qualitative Toxicological Evaluation of Thiol-ene “click” Chemistry-based Polyanhydrides and Their Degradation Products. J. Biomed. Mater. Res., Part A 2016, 104, 1936–1945. 10.1002/jbm.a.35724. 27012532

[ref92] Geraili A.; Mequanint K. Systematic Studies on Surface Erosion of Photocrosslinked Polyanhydride Tablets and Data Correlation with Release Kinetic Models. Polymers. 2020, 12, 1105 10.3390/polym12051105. 32408683 PMC7285269

[ref93] Bian L.; Mohammed H. S.; Shipp D. A.; Goulet P. J. G. Raman Microspectroscopy Study of the Hydrolytic Degradation of Polyanhydride Network Polymers. Langmuir. 2019, 35, 6387–6392. 10.1021/acs.langmuir.8b04334. 30998022

[ref94] Snyder B. L.; Mohammed H. S.; Samways D. S. K.; Shipp D. A. Drug Delivery and Drug Efficacy from Amorphous Poly(thioether anhydrides). Macromol. Biosci. 2020, 20, 1900377 10.1002/mabi.201900377. 32207234

[ref95] Mohammed H. S.; Samways D. S. K.; Shipp D. A. Cellular Delivery of Hoechst 33342 Anticancer Drug from Crosslinked Poly(thioether anhydrides): A Cytotoxicity and Efficacy Study. Polymers in Therapeutic Delivery; Fujiwara T., Liu X. M., Ohya Y., Wang Y., Eds.; ACS Symposium Series; American Chemical Society, 2020; Vol. 1350, pp 64–77. DOI: 10.1021/bk-2020-1350.ch006.

[ref96] Dasgupta S.; Mondal S.; Ray S.; Singh Y. P.; Maji K. Hydroxyapatite-collagen Nanoparticles Reinforced Polyanhydride Based Injectable Paste for Bone Substitution: Effect of Dopant Addition in Vitro. J. Biomater. Sci., Polym. Ed. 2021, 32, 1312–1336. 10.1080/09205063.2021.1916867. 33874849

[ref97] Cheng G.; Aponte M. A.; Ramírez C. A. Cross-linked Amino Acid-containing Polyanhydrides for Controlled Drug Release Applications. Polymer. 2004, 45, 3157–3162. 10.1016/j.polymer.2004.03.035.

[ref98] Mönkäre J.; Hakala R. A.; Vlasova M. A.; Huotari A.; Kilpeläinen M.; Kiviniemi A.; Meretoja V.; Herzig K. H.; Korhonen H.; Seppälä J. V.; Järvinen K. Biocompatible Photo Crosslinked Poly(ester anhydride) Based on Functionalized Poly (ε-caprolactone) Prepolymer Shows Surface Erosion Controlled Drug Release In Vitro and In Vivo. J. Controlled Release 2010, 146, 349–355. 10.1016/j.jconrel.2010.06.005. 20558218

[ref99] Dong A.; Zhang T. Paclitaxel Release from Polyether-Anhydrides Prepared with UV-Curing Process. Int. J. Polym. Sci. 2013, 916571, 1–6. 10.1155/2013/916571.

[ref100] Anseth K. S.; Shastri V. R.; Langer R. Photopolymerizable Degradable Polyanhydrides With Osteocompatibility. Nat. Biotechnol. 1999, 17, 156–159. 10.1038/6152. 10052351

[ref101] Muggli D. S.; Burkoth A. K.; Anseth K. S. Crosslinked Polyanhydrides for Use in Orthopaedic Applications: Degradation Behaviour and Mechanics. J. Biomed. Mater. Res. 1999, 46, 271–278. 10.1002/(SICI)1097-4636(199908)46:2<271::AID-JBM17>3.0.CO;2-X. 10380006

[ref102] Anseth K. S.; Svaldi D. C.; Laurencin C. T.; Langer R. Photopolymerization of Novel Degradable Networks for Orthopedic Applications. Photopolymerization; Scranton A. B., Bowman C. N., Peiffer R. W., Eds.; ACS Symposium Series, Vol. 673; American Chemical Society, 1997; pp 189–202. DOI: 10.1021/bk-1997-0673.ch014.

[ref103] Chu I. M.; Liu T. H.; Chen Y. R. Preparation and Characterization of Sustained Release System Based on Polyanhydride Microspheres with Core/shell-like Structures. J. Polym. Res. 2019, 26, 1 10.1007/s10965-018-1657-5.

[ref104] Domb A. J.; Gallardo C. F.; Langer R. Poly(anhydrides). 3. Poly(anhydrides)Based on Aliphatic-Aromatic Diacids. Macromolecules. 1989, 22, 3200–3204. 10.1021/ma00198a002.

[ref105] Teomim D.; Domb A. J. Fatty acid Terminated Polyanhydrides. J. Polym. Sci., Part A: Polym. Chem. 1999, 37, 3337–3344. 10.1002/(SICI)1099-0518(19990815)37:16<3337::AID-POLA32>3.0.CO;2-S.

[ref106] Teomim D.; Domb A. J. Nonlinear Fatty Acid Terminated Polyanhydrides. Biomacromolecules. 2001, 2, 37–44. 10.1021/bm000081r. 11749153

[ref107] Domb A. J.; Maniar M. Absorbable Biopolymers Derived from Dimer Fatty Acids. J. Polym. Sci., Part A: Polym. Chem. 1993, 31, 1275 10.1002/pola.1993.080310523.

[ref108] Park E. S.; Maniar M.; Shah J. C. Biodegradable Polyanhydride Devices of Cefazolin Sodium, Bupivacaine, and Taxol for Local Drug Delivery: Preparation, and Kinetics and Mechanism of In Vitro Release. J. Controlled Release 1998, 52, 179–189. 10.1016/S0168-3659(97)00223-X. 9685948

[ref109] Teomim D.; Fishbien I.; Golomb G.; Orloff L.; Mayberg M.; Domb A. J. Perivascular Delivery of Heparin for the Reduction of Smooth Muscle Cell Proliferation After Endothelial Injury. J. Controlled Release 1999, 60, 129–142. 10.1016/S0168-3659(99)00071-1. 10370177

[ref110] Teomim D.; Nyska A.; Domb A. J. Ricinoleic acid-based Biopolymers. J. Biomed. Mater. Res. 1999, 45, 258–267. 10.1002/(SICI)1097-4636(19990605)45:3<258::AID-JBM14>3.0.CO;2-W. 10397984

[ref111] Domb A. J.; Nudelman R. Biodegradable Polymers Derived from Natural Fatty Acids. J. Polym. Sci., Part A: Polym. Chem. 1995, 33, 717–725. 10.1002/pola.1995.080330413.

[ref112] Domb A. J.; Ron E.; Langer R. Poly (anhydrides). 2. One-Step Polymerization Using Phosgene or Diphosgene as Coupling Agents. Macromolecules. 1988, 21, 1925–1929. 10.1021/ma00185a008.

[ref113] Lundmark S.; SjÖling M.; Albertsson A. C. Polymerization of Oxepan-2,7-dione in Solution and Synthesis of Block Copolymers of Oxepan-2,7-dione and 2-Oxepanone. J. Macromol. Sci., Chem. 1991, 28, 15–29. 10.1080/00222339108052083.

[ref114] Albertsson A. C.; Lundmark S. Synthesis of Poly(Adipic Anhydride) by Use of Ketene. J. Macromol. Sci., Chem. 1988, 25, 247–258. 10.1080/00222338808051969.

[ref115] Tarcha P. J.; Su L.; Baker T.; Langridge D.; Shastri V.; Langer R. Stability of Photocurable Anhydrides: Methacrylic acid Mixed Aanhydrides of Nontoxic Diacids. J. Polym. Sci., Part A: Polym. Chem. 2001, 39, 4189–4195. 10.1002/pola.10073.

[ref116] Domanskyi S.; Poetz K. L.; Shipp D. A.; Privman V. Reaction-diffusion Degradation Model for Delayed Erosion of Cross-linked Polyanhydride Biomaterials. Phys. Chem. Chem. Phys. 2015, 17, 13215–13222. 10.1039/C5CP00473J. 25766671

[ref117] Sajjad H.; Lillie L. M.; Lau C. M.; Ellison C. J.; Tolman W. B.; Reineke T. M. Degradable polyanhydride networks derived from itaconic acid. Polym. Chem. 2021, 12, 608–617. 10.1039/D0PY01388A.

[ref118] Stebbins N. D.; Faig J. J.; Yu W.; Guliyev R.; Uhrich K. E. Polyactives: Controlled and Sustained Bioactive Release via Hydrolytic Degradation. Biomater. Sci. 2015, 3, 1171–1187. 10.1039/C5BM00051C. 26222033 PMC4519997

[ref119] Park E.-S.; Maniar M.; Shah J. Effects of Model Compounds with Varying Physicochemical Properties on Erosion of Polyanhydride Devices. J. Controlled Release 1996, 40, 111–121. 10.1016/0168-3659(95)00182-4.

[ref120] Zhang T.; Gu M.; Yu X. Degradation and Drug Delivery Properties of Poly(1,4-cyclohexanedicarboxylic anhydride). J. Biomater. Sci., Polym. Ed. 2001, 12, 491–501. 10.1163/156856201300194234. 11469780

[ref121] Albertsson A. C.; Liu Y. Comparison Between Physical Blending and Copolymerization of Poly(trimethylene carbonate) and Poly(adipic anhydride) with Special Regard to Compatibility, Morphology and Degradation. J. Macromol. Sci., Part A: Pure Appl.Chem. 1997, 34 (8), 1457–1482. 10.1080/10601329708011056.

[ref122] Deng Z.; Riga E. K.; Lienkamp K. Degradable Polymer Films Made from Poly(salicylic-acid-co-sebacic acid) and Poly(sebacic anhydride)/Poly(adipic anhydride) Blends: Degradation Kinetics and Use as Sacrificial Layers for Polymer Multilayer Systems. Macromol. Chem. Phys. 2020, 221, 2000106 10.1002/macp.202000106. 34646086 PMC7611817

[ref123] Burkersroda F. V.; Schedl L.; Gopferich A. Why Degradable Polymers Undergo Surface Erosion or Bulk Erosion. Biomaterials. 2002, 23, 4221–4231. 10.1016/S0142-9612(02)00170-9. 12194525

[ref124] Deng Z.; Schweigerdt A.; Norow A.; Lienkamp K. Degradation of Polymer Films on Surfaces: A Model Study with Poly(sebacic anhydride). Macromol. Chem. Phys. 2019, 220, 1900121 10.1002/macp.201900121. 34404980 PMC7611508

[ref125] Yin L.; Huang X.; Xu H.; Zhang Y.; Lam J.; Cheng J.; Rogers J. A. Materials, Designs, and Operational Characteristics for Fully Biodegradable Primary Batteries. Adv. Mater. 2014, 26, 3879–3884. 10.1002/adma.201306304. 24652717

[ref126] Kang S. K.; Murphy R. K. J.; Hwang S. W.; Lee S. M.; Harburg D. V.; Krueger N. A.; Shin J.; Gamble P.; Cheng H.; Yu S.; Liu Z.; Mc Call J. G.; Stephen M.; Ying H.; Kim J.; Park G.; Webb R. C.; Lee C. H.; Chung S.; Wie D. S.; Gujar A. D.; Vemulapalli B.; Kim A. H.; Lee K. M.; Cheng J.; Huang Y.; Lee S. H.; Braun P. V.; Ray W. Z.; Rogers J. A. Bioresorbable Silicon Electronic Sensors for the Brain. Nature. 2016, 530, 71–79. 10.1038/nature16492. 26779949

[ref127] Shim J. S.; Rogers J. A.; Kang S. K. Physically Transient Electronic Materials and Devices. Mater. Sci. Eng., R. 2021, 145, 100624 10.1016/j.mser.2021.100624.

[ref128] Gao Y.; Zhang Y.; Wang X.; Sim K.; Liu J.; Chen J.; Feng X.; Xu H.; Yu C. Moisture-triggered Physically Transient Electronics. Sci. Adv. 2017, 3, e1701222 10.1126/sciadv.1701222. 28879237 PMC5580884

[ref129] Choi Y. S.; Koo J.; Lee Y. J.; Lee G.; Avila R.; Ying H.; Reeder J.; Hambitzer L.; Im K.; Kim J.; Lee K. M.; Cheng J.; Huang Y.; Kang S. K.; Rogers J. A. Biodegradable Polyanhydrides as Encapsulation Layers for Transient Electronics. Adv. Funct. Mater. 2020, 30, 2000941 10.1002/adfm.202000941.

[ref130] Kelly S. M.; Mitra A.; Mathur S.; Narasimhan B. Synthesis and Characterization of Rapidly Degrading Polyanhydrides as Vaccine Adjuvants. ACS Biomater. Sci. Eng. 2020, 6, 265–276. 10.1021/acsbiomaterials.9b01427. 33463223

[ref131] Narasimhan B.; Goodman J. T.; Ramirez J. E. V. Rational Design of Targeted Next-Generation Carriers for Drug and Vaccine Delivery. Annu. Rev. Biomed. Eng. 2016, 18, 25–49. 10.1146/annurev-bioeng-082615-030519. 26789697

[ref132] Mullis A. S.; Jacobson S. J.; Narasimhan B. High-Throughput Synthesis and Screening of Rapidly Degrading Polyanhydride Nanoparticles. ACS Comb. Sci. 2020, 22, 172–183. 10.1021/acscombsci.9b00162. 32125826

[ref133] Grego E. A.; Siddoway A. C.; Uz M.; Liu L.; Christiansen J. C.; Ross K. A.; Kelly S. M.; Mallapragada S. K.; Wannemuehler M. J.; Narasimhan B. Polymeric Nanoparticle-Based Vaccine Adjuvants and Delivery Vehicles. Curr. Top. Microbiol. Immunol. 2020, 433, 29–76. 10.1007/82_2020_226. PMC8107186 33165869

[ref134] Goodman J. T.; Mullis A. S.; Dunshee L.; Mitra A.; Narasimhan B. Automated High-Throughput Synthesis of Protein-Loaded Polyanhydride Nanoparticle Libraries. ACS Comb. Sci. 2018, 20, 298–307. 10.1021/acscombsci.8b00008. 29617113

[ref135] Mallapragada S. K.; Narasimhan B. Immunomodulatory Biomaterials. Int. J. Pharm. 2008, 364, 265–271. 10.1016/j.ijpharm.2008.06.030. 18662761

[ref136] Ulery B. D.; Kumar D.; Ramer-Tait A. E.; Metzger D. W.; Wannemuehler M. J.; et al. Design of a Protective Single-Dose Intranasal Nanoparticle-Based Vaccine Platform for Respiratory Infectious Diseases. PLoS One. 2011, 6, e17642 10.1371/journal.pone.0017642. 21408610 PMC3048296

[ref137] Wagner-Muniz D. A.; Haughney S. L.; Kelly S. M.; Wannemuehler M. J.; Narasimhan B. Room Temperature Stable PspA-Based Nanovaccine Induces Protective Immunity. Front. Immunol. 2018, 9, 325 10.3389/fimmu.2018.00325. 29599766 PMC5863507

[ref138] Haughney S. L.; Ross K. A.; Boggiatto P. M.; Wannemuehler M. J.; Narasimhan B. Effect of Nanovaccine Chemistry on Humoral Immune Response Kinetics and Maturation. Nanoscale. 2014, 6, 13770–13778. 10.1039/C4NR03724C. 25285425

[ref139] Ross K.; Senapati S.; Alley J.; Darling R.; Goodman J.; Jefferson M.; Uz M.; Guo B.; Yoon K. J.; Verhoeven D.; Kohut M.; Mallapragada S.; Wannemuehler M.; Narasimhan B. Single Dose Combination Nanovaccine Provides Protection Against Influenza A Virus in Young and Aged Mice. Biomater. Sci. 2019, 7, 809–821. 10.1039/C8BM01443D. 30663733

[ref140] Ross K.; Loyd H.; Wu W.; Huntimer L.; Ahmed S.; Sambol A.; Broderick S.; Flickinger Z.; Rajan K.; Bronich T.; Mallapragada S.; Wannemuehler M.; Carpenter S.; Narasimhan B. Hemagglutinin-based Polyanhydride Nanovaccines Against H5N1 Influenza Elicit Protective Virus Neutralizing Titers and Cell-Mediated Immunity. Int. J. Nanomed. 2014, 10, 229–243. 10.2147/IJN.S72264. PMC4284014 25565816

[ref141] Huntimer L. M.; Ross K. A.; Darling R. J.; Winterwood N. E.; Boggiatto P.; Narasimhan B.; Ramer-Tait A. E.; Wannemuehler M. J. Polyanhydride Nanovaccine Platform Enhances Antigen-specific Cytotoxic T Cell Responses. Technology. 2014, 2, 171–175. 10.1142/S2339547814500162.

[ref142] Vela Ramirez J.; Tygrett L.; Hao J.; Habte H.; Cho M.; Greenspan N.; Waldschmidt T.; Narasimhan B. Polyanhydride Nanovaccines Induce Germinal Center B Cell Formation and Sustained Serum Antibody Responses. J. Biomed. Nanotechnol. 2016, 12, 1303–1311. 10.1166/jbn.2016.2242. 27319223 PMC5438750

[ref143] Ulery B. D.; Kumar D.; Ramer-Tait A. E.; Metzger D. W.; Wannemuehler M. J.; Narasimhan B. Design of a Protective Single-Dose Intranasal Nanoparticle-Based Vaccine Platform for Respiratory Infectious Diseases. PLoS One. 2011, 6, e17642 10.1371/journal.pone.0017642. 21408610 PMC3048296

[ref144] Ross K.; Adams J.; Loyd H.; Ahmed S.; Sambol A.; Broderick S.; Rajan K.; Kohut M.; Bronich T.; Wannemuehler M. J.; Carpenter S.; Mallapragada S.; Narasimhan B. Combination Nanovaccine Demonstrates Synergistic Enhancement in Efficacy against Influenza. ACS Biomater. Sci. Eng. 2016, 2, 368–374. 10.1021/acsbiomaterials.5b00477. 33429541

[ref145] McGill J. L.; Kelly S. M.; Kumar P.; et al. Efficacy of Mucosal Polyanhydride Nanovaccine Against Respiratory Syncytial Virus Infection in the Neonatal Calf. Sci. Rep. 2018, 8, 3021 10.1038/s41598-018-21292-2. 29445124 PMC5813012

[ref146] Petersen A. K.; Ramer-Tait A. E.; Broderick S. R.; Kong C. S.; Ulery B. D.; Rajan K.; Wannemuehler M. J.; Narasimhan B. Activation of Innate Immune Responses in a Pathogen-mimicking Manner by Amphiphilic Polyanhydride Nanoparticle Adjuvants. Biomaterials. 2011, 32, 6815–6822. 10.1016/j.biomaterials.2011.05.063. 21703679

[ref147] Phanse Y.; Lueth P.; Tait A. E. R.; Conde B. R. C.; Wannemuehler M. J.; Narasimhan B.; Bellaire B. H. Cellular Internalization Mechanisms of Polyanhydride Particles: Implications for Rational Design of Drug Delivery Vehicles. J. Biomed. Nanotechnol. 2016, 12, 1544–1552. 10.1166/jbn.2016.2259. 29337493

[ref148] Huntimer L.; Ramer-Tait A. E.; Petersen L. K.; Ross K. A.; Walz K. A.; Wang C.; Hostetter J.; Narasimhan B.; Wannemuehler M. J. Evaluation of Biocompatibility and Administration Site Reactogenicity of Polyanhydride-Particle-Based Platform for Vaccine Delivery. Adv. Healthcare Mater. 2013, 2, 369–378. 10.1002/adhm.201200181. 23184561

[ref149] Kipper M. J.; Wilson J. H.; Wannemuehler M. J.; Narasimhan B. Single Dose Vaccine Based on Biodegradable Polyanhydride Microspheres Can Modulate Immune Response Mechanism. J. Biomed. Mater. Res., Part A 2006, 76A, 798–810. 10.1002/jbm.a.30545. 16345084

[ref150] Dhakal S.; Goodman J.; Bondra K.; Lakshmanappa Y. S.; Hiremath J.; Shyu D. L.; Ouyang K.; Kang K. il; Krakowka S.; Wannemuehler M. J.; et al. Polyanhydride Nanovaccine Against Swine Influenza Virus in Pigs. Vaccine. 2017, 35, 1124–1131. 10.1016/j.vaccine.2017.01.019. 28117173

[ref151] Dhakal S.; Ghimire S.; Renu S.; Ross K. A.; Lakshmanappa Y. S.; Hogshead B. T.; Bernardo P.; Lee C. W.; Wannemuehler M. J.; Narasimhan B.; Renukaradhya G. J. Evaluation of CpG-ODN-adjuvanted Polyanhydride-based Intranasal Influenza Nanovaccine in Pigs. Vet. Microbiol. 2019, 237, 108401 10.1016/j.vetmic.2019.108401. 31585639

[ref152] Siddoway L. C.; Verhoeven D.; Ross K. A.; Wannemuehler M. J.; Mallapragada S. K.; Narasimhan B. Structural Stability and Antigenicity of Universal Equine H3N8 Hemagglutinin Trimer upon Release from Polyanhydride Nanoparticles and Pentablock Copolymer Hydrogels. ACS Biomater. Sci. Eng. 2022, 8, 2500–2507. 10.1021/acsbiomaterials.2c00219. 35604784 PMC12108903

[ref153] Ross K.; Senapati S.; Alley J.; Darling R.; Goodman J.; Jefferson M.; Uz M.; Guo B.; Yoon K. J.; Verhoeven D.; Kohut M.; Mallapragada S.; Wannemuehlerc M.; Narasimhan B. Single Dose Combination Nanovaccine Provides Protection Against Influenza A Virus in Young and Aged Mice. Biomater. Sci. 2019, 7, 809–821. 10.1039/C8BM01443D. 30663733

[ref154] Kelly S. M.; Larsen K. R.; Darling R.; Petersen A. C.; Bellaire B. H.; Wannemuehler M. J.; Narasimhan B. Single-dose Combination Nanovaccine Induce Both Rapid and Durable Humoral Immunity and Toxin Neutralizing Antibody Response Against Bacillus Anthracis. Vaccine. 2021, 39 (29), 3862–3870. 10.1016/j.vaccine.2021.05.077. 34090702 PMC8325489

[ref155] Liu L.; Kshirsagar P.; Christiansen J.; Gautam S. K.; Aithal A.; Gulati M.; Kumar S.; Solheim J. C.; Batra S. K.; Jain M.; Wannemuehler M. J.; Narasimhan B. Polyanhydride Nanoparticles Stabilize Pancreatic Cancer Antigen MUC4β. J. Biomed. Mater. Res. 2021, 109, 893–902. 10.1002/jbm.a.37080. PMC8100985 32776461

[ref156] Banerjee K.; Gautam S. K.; Kshirsagar P.; Ross K. A.; Spagnol G.; Sorgen P.; Wannemuehler M. J.; Narasimhan B.; Solheim J. C.; Kumar S.; Batra S. K.; Jain M. Amphiphilic polyanhydride-based recombinant MUC4β-nanovaccine activates dendritic cells. Genes Cancer. 2019, 10 (3–4), 52–62. 10.18632/genesandcancer.189. 31258832 PMC6584211

[ref157] Kingstad-Bakke B. A.; Chandrasekar S. S.; Phanse Y.; Ross K. A.; Hatta M.; Suresh M.; Kawaoka Y.; Osorio J. E.; Narasimhan B.; Talaat A. M. Effective Mosaic-based Nanovaccines Against Avian Influenza in Poultry, Vaccine. 2019, 37, 5051–5058. 10.1016/j.vaccine.2019.06.077. 31300285

[ref158] Thukral A.; Ross K.; Hansen C.; Phanse Y.; Narasimhan B.; Steinberg H.; Talaat A. M. A Single Dose Polyanhydride-Based Nanovaccine against Paratuberculosis Infection. npj Vaccines. 2020, 15, 1–10. 10.1038/s41541-020-0164-y. PMC7021715 32128256

[ref159] Chiu Li L.; Deng J.; Stephens D. Polyanhydride Implant for Antibiotic Delivery - From the Bench to the Clinic. Adv. Drug Delivery Rev. 2002, 54, 963–986. 10.1016/S0169-409X(02)00053-4. 12384317

[ref160] Masters D. B.; Berde C. B.; Dutta S.; Turek T.; Langer R. Sustained Local Anesthetic Release from Bioerodible Polymer Matrices: A Potential Method for Prolonged Regional Anesthesia. Pharm. Res. 1993, 10, 1527–1532. 10.1023/A:1018995913972. 8272418

[ref161] Jia F.; Liu X.; Li L.; Mallapragada S.; Narasimhan B.; Wang Q. Multifunctional Nanoparticles for Targeted Delivery of Immune Activating and Cancer Therapeutic Agents. J. Controlled Release 2013, 172, 1020–1034. 10.1016/j.jconrel.2013.10.012. 24140748

[ref162] Bagherifam S.; Griffiths G. W.; Maelandsmo G. M.; Nyström B.; Hasirci V.; Hasirci N.; Nyströ B. Micro and Nano Carriers Poly(Sebacic Anhydride) Nanocapsules as Carriers: Effects of Preparation Parameters on Properties and Release of Doxorubicin Poly(Sebacic Anhydride) Nanocapsules as Carriers: Effects of Preparation Parameters on Properties and Release of Doxorubicin. J. Microencapsulation. 2015, 32, 166–174. 10.3109/02652048.2014.973073. 25323326

[ref163] Quick D. J.; Macdonald K. K.; Anseth K. S. Delivering DNA from Photocrosslinked, Surface Eroding Polyanhydrides. J. Controlled Release 2004, 97, 333–343. 10.1016/j.jconrel.2004.03.001. 15196760

[ref164] Kubek M. J.; Liang D.; Byrd K. E.; Domb A. J. Prolonged Seizure Suppression by a Single Implantable Polymeric-TRH Microdisk Preparation. Brain Res. 1998, 809, 189–197. 10.1016/S0006-8993(98)00860-9. 9853110

[ref165] Carrillo-Conde B. R.; Darling R. J.; Seiler S. J.; Ramer-Tait A. E.; Wannemuehler M. J.; Narasimhan B. Sustained Release and Stabilization of Therapeutic Antibodies Using Amphiphilic Polyanhydride Nanoparticles. Chem. Eng. Sci. 2015, 125, 98–107. 10.1016/j.ces.2014.08.015.

[ref166] Haughney S. L.; Petersen L. K.; Schoofs A. D.; Ramer-Tait A. E.; King J. D.; Briles D. E.; Wannemuehler M. J.; Narasimhan B. Retention of Structure, Antigenicity, and Biological Function of Pneumococcal Surface Protein A (PspA) Released from Polyanhydride Nanoparticles. Acta Biomater. 2013, 9, 8262–8271. 10.1016/j.actbio.2013.06.006. 23774257 PMC3777629

[ref167] Rosario-Meléndez R.; Ouimet M. A.; Uhrich K. E. Formulation of Salicylate-Based Poly(Anhydride-Ester) Microspheres for Short-and Long-Term Salicylic Acid Delivery. Polym. Bull. 2013, 70, 343–351. 10.1007/s00289-012-0839-2. PMC3571728 23420391

[ref168] Delgado-Rivera R.; Rosario-Mel Endez R.; Yu W.; Uhrich K. E. Biodegradable Salicylate-Based Poly(Anhydride-Ester) Microspheres for Controlled Insulin Delivery. J. Biomed. Mater. Res., Part A 2014, 102, 2736–2742. 10.1002/jbm.a.34949. PMC3952025 24027012

[ref169] Jaszcz K. Chinese Journal of Polymer Science Highly Porous Crosslinked Poly(Ester-Anhydride) Microspheres with High Loading Efficiency. Chin. J. Polym. Sci. 2015, 33, 1271–1282. 10.1007/s10118-015-1677-0.

[ref170] Jaszcz K. Effect of Basic Factors of Preparation on Characteristics, Hydrolytic Degradation, and Drug Release From Poly(Ester-Anhydride) Microspheres. Int. J. Polym. Mater. Polym. Biomater. 2014, 63, 97–106. 10.1080/00914037.2013.769254.

[ref171] Jaszcz K.; Łukaszczyk J. Epoxidation of Pendant Allyl Groups in Poly(Ester-Anhydride)s Proposed for Application in Drug Delivery. React. Funct. Polym. 2012, 72, 650–656. 10.1016/j.reactfunctpolym.2012.06.013.

[ref172] Lopac S. K.; Torres M. P.; Wilson-Welder J. H.; Wannemuehler M. J.; Narasimhan B. Effect of Polymer Chemistry and Fabrication Method on Protein Release and Stability from Polyanhydride Microspheres. J. Biomed. Mater. Res., Part B Appl. Biomater. 2009, 91B, 938–947. 10.1002/jbm.b.31478. PMC3710783 19642209

[ref173] Alakurtti S.; Mäkelä T.; Koskimies S.; Yli-Kauhaluoma J. Pharmacological Properties of the Ubiquitous Natural Product Betulin. Eur. J. Pharm. Sci. 2006, 29, 1–13. 10.1016/j.ejps.2006.04.006. 16716572

[ref174] Zhou S.; Sun W.; Zhai Y. Amphiphilic Block Copolymer NPs Obtained by Coupling Ricinoleic Acid/Sebacic Acids and MPEG: Synthesis, Characterization, and Controlled Release of Paclitaxel. J. Biomater. Sci., Polym. Ed. 2018, 29, 2201–2217. 10.1080/09205063.2018.1532136. 30285542

[ref175] Zhao A.; Zhou S.; Zhou Q.; Chen T. Thermosensitive Micelles from PEG-Based Ether-Anhydride Triblock Copolymers. Pharm. Res. 2010, 27, 1627–1643. 10.1007/s11095-010-0155-1. 20428931

[ref176] Wang J.; Yang G.; Guo X.; Tang Z.; Zhong Z.; Zhou S. Redox-Responsive Polyanhydride Micelles for Cancer Therapy. Biomaterials. 2014, 35, 3080–3090. 10.1016/j.biomaterials.2013.12.025. 24388799

[ref177] Prudencio A.; Faig J. J.; Song M.; Uhrich K. E. Phenolic Acid-Based Poly(Anhydride-Esters) as Antioxidant Biomaterials. Macromol. Biosci. 2016, 16, 214–222. 10.1002/mabi.201500244. 26425923 PMC4752411

[ref178] Carbone-Howell A. L.; Stebbins N. D.; Uhrich K. E. Poly(Anhydride-Esters) Comprised Exclusively of Naturally Occurring Antimicrobials and EDTA: Antioxidant and Antibacterial Activities. Biomacromolecules. 2014, 15, 1889–1895. 10.1021/bm500303a. 24702678 PMC4020595

[ref179] Ouimet M. A.; Griffin J.; Carbone-Howell A. L.; Wu W. H.; Stebbins N. D.; Di R.; Uhrich K. E. Biodegradable Ferulic Acid-Containing Poly(Anhydride-Ester): Degradation Products with Controlled Release and Sustained Antioxidant Activity. Biomacromolecules. 2013, 14, 854–861. 10.1021/bm3018998. 23327626 PMC3595371

[ref180] Ouimet M. A.; Stebbins N. D.; Uhrich K. E. Biodegradable Coumaric acid-based Poly(anhydride-ester) Synthesis and Subsequent Controlled Release. Macromol. Rapid Commun. 2013, 34, 1231–6. 10.1002/marc.201300323. 23836606 PMC3789234

[ref181] Schmeltzer R. C.; Uhrich K. E. Synthesis and Characterization of Antiseptic-Based Poly(Anhydride-Esters). Polym. Bull. 2006, 57, 281–291. 10.1007/s00289-006-0561-z. PMC3769797 24039323

[ref182] Stebbins N. D.; Yu W.; Uhrich K. E. Linear, Mannitol-Based Poly(Anhydride-Esters) with High Ibuprofen Loading and Anti-Inflammatory Activity. Biomacromolecules. 2015, 16, 3632–3639. 10.1021/acs.biomac.5b01088. 26450447

[ref183] Lee Y. S.; Griffin J.; Masand S. N.; Shreiber D. I.; Uhrich K. E. Salicylic Acid-Based Poly(Anhydride-Ester) Nerve Guidance Conduits: Impact of Localized Drug Release on Nerve Regeneration. J. Biomed. Mater. Res., Part A 2016, 104, 975–982. 10.1002/jbm.a.35630. 26691691

[ref184] Prudencio A.; Carbone A. L.; Griffin J.; Uhrich K. E. A Novel Approach for Incorporation of Mono-Functional Bioactive Phenols into Polyanhydrides. Macromol. Rapid Commun. 2009, 30, 1101–1108. 10.1002/marc.200900059. 21706573

[ref185] Prudencio A.; Stebbins N. D.; Johnson M.; Song M.; Langowski B. A.; Uhrich K. E. Polymeric Prodrugs of Ampicillin as Antibacterial Coatings. J. Bioact. Compat. Polym. 2014, 29, 208–220. 10.1177/0883911514528410.

[ref186] Johnson M. L.; Uhrich K. E. Concurrent Release of Admixed Antimicrobials and Salicylic Acid from Salicylate-Based Poly(Anhydride-Esters). J. Biomed. Mater. Res., Part A 2009, 91A, 671–678. 10.1002/jbm.a.32288. PMC2767466 19180627

[ref187] Dasgupta Q.; Chatterjee K.; Madras G. Controlled Release of Salicylic Acid from Biodegradable Cross-Linked Polyesters. Mol. Pharmaceutics. 2015, 12, 3479–3489. 10.1021/acs.molpharmaceut.5b00515. 26284981

[ref188] Chandorkar Y.; Bhagat R. K.; Madras G.; Basu B. Cross-Linked, Biodegradable, Cytocompatible Salicylic Acid Based Polyesters for Localized, Sustained Delivery of Salicylic Acid: An In Vitro Study. Biomacromolecules. 2014, 15, 863–875. 10.1021/bm401715z. 24517727

[ref189] Bhaskar N.; Padmavathy N.; Jain S.; Bose S.; Basu B. Modulated in Vitro Biocompatibility of a Unique Cross-Linked Salicylic Acid–Poly(ε-Caprolactone)-Based Biodegradable Polymer. ACS Appl. Mater. Interfaces. 2016, 8, 29721–29733. 10.1021/acsami.6b10711. 27726328

[ref190] Dasgupta Q.; Chatterjee K.; Madras G. Physical Insights into Salicylic acid Release from Poly(anhydrides). Phys. Chem. Chem. Phys. 2016, 18, 2112–2119. 10.1039/C5CP06858D. 26689269

